# Pneumococcal capsule expression is controlled through a conserved, distal *cis*-regulatory element during infection

**DOI:** 10.1371/journal.ppat.1011035

**Published:** 2023-01-31

**Authors:** David G. Glanville, Ozcan Gazioglu, Michela Marra, Valerie L. Tokars, Tatyana Kushnir, Medhanie Habtom, Nicholas J. Croucher, Yaffa Mizrachi Nebenzahl, Alfonso Mondragón, Hasan Yesilkaya, Andrew T. Ulijasz

**Affiliations:** 1 Department of Microbiology and Immunology, Loyola University Chicago, Maywood, Illinois, United States of America; 2 Department of Respiratory Sciences, University of Leicester, University Road, Leicester, United Kingdom; 3 Department of Pharmacology, Feinberg School of Medicine, Northwestern University, Chicago, Illinois, United States of America; 4 The Shraga Segal Department of Microbiology, Immunology and Genetics, Faculty of Health Sciences, Ben-Gurion University of The Negev, Beer-Sheva, Israel; 5 MRC Centre for Global Infectious Disease Analysis, Department of Infectious Disease Epidemiology, Sir Michael Uren Hub, Imperial College London, London, United Kingdom; 6 Department of Molecular Biosciences, Northwestern University, Evanston, Illinois, United States of America; The University of Alabama at Birmingham, UNITED STATES

## Abstract

*Streptococcus pneumoniae* (the pneumococcus) is the major cause of bacterial pneumonia in the US and worldwide. Studies have shown that the differing chemical make-up between serotypes of its most important virulence factor, the capsule, can dictate disease severity. Here we demonstrate that control of capsule synthesis is also critical for infection and facilitated by two broadly conserved transcription factors, SpxR and CpsR, through a distal *cis*-regulatory element we name the *37-CE*. Strikingly, changing only three nucleotides within this sequence is sufficient to render pneumococcus avirulent. Using *in vivo* and *in vitro* approaches, we present a model where SpxR interacts as a unique trimeric quaternary structure with the *37-CE* to enable capsule repression in the airways. Considering its dramatic effect on infection, variation of the *37-CE* between serotypes suggests this molecular switch could be a critical contributing factor to this pathogen’s serotype-specific disease outcomes.

## Author summary

*Streptococcus pneumoniae* (the pneumococcus) is a Gram-positive pathogen that causes significant morbidity and mortality in the US and worldwide. The pneumococcus first colonizes the upper respiratory tract asymptomatically, and then drops into the lung to cause pneumonia, followed by sepsis (blood infection), where patient mortality rates exceed 20%. Studies have indicated that the reason *S*. *pneumoniae* can survive in the blood so well is due to its protective polysaccharide capsule. This vital virulence factor acts as a sugary coat which shields the bacterium from immune detection, opsonization, and phagocytosis. However, how this metabolically expensive virulence factor is regulated during colonization, lung infection, and sepsis has remained enigmatic. Here we describe two transcription factors, SpxR and CpsR, which repress capsule biosynthesis in the airways through a small, conserved, regulatory piece of DNA (the *37-CE*) until the onset of sepsis–where repression is relieved, allowing for increased capsule biosynthesis, and by association, the high mortality associated with this condition. The *37-CE* varies considerably among the 100+ pneumococcal serotypes, each of which has a chemically-distinct capsule composition. This may, in part, explain the variation in serotype-dependent disease severity we have observed for decades.

## Introduction

Prior to infection, *S*. *pneumoniae* (the pneumococcus), a human-specific pathogen, asymptomatically colonizes the nasopharynx. To cause pneumonia, the pneumococcus must disseminate from the upper respiratory tract into the lung [1,2]. From the lung, it can then enter the blood where it may colonize other tissues, such as the heart or central nervous system to cause cardiac damage [3] or meningitis, respectively [4]. Although the transition from commensal to invasive pathogen is a largely enigmatic process, it is presently understood that this switch is facilitated by regulation of capsule expression [5–8]. In support, *in vitro* and *in vivo* experiments have suggested that during colonization the capsule is diminished to allow adherence to the nasopharyngeal epithelium and, conversely, expands during sepsis to avoid phagocytic killing [6,9]. Deletion of the capsule prevents the pneumococcus from causing life-threatening infections, further highlighting the importance of this major virulence factor [10,11]. Therefore, understanding the regulatory mechanisms controlling pneumococcal capsule expression could pave the way for novel therapies.

In all but two of the 100+ known antigenically-distinct *S*. *pneumoniae* serotypes [12], the capsule is synthesized via a conserved Wzy polymerase-dependent mechanism whose genes are encoded within a single operon (the *cps* locus) [13]. It is thought that capsule diversity, size and its regulation play important roles in dictating a serotype’s ability to colonize or become invasive; characteristics that vary considerably both between and even within specific serotypes [14,15]. Interestingly, despite the enormous diversity within the *cps* locus, the upstream regulatory DNA is highly similar between serotypes [16], suggesting conserved regulatory mechanisms. Although much has been accomplished in identifying how enzymes assemble the capsule biochemically [13] and covalently attach it to the cell surface [17], few reports have lent insight into how the *cps* locus is transcriptionally regulated to facilitate infection [5,18–20]. Our data presented here show how two conserved transcription factors, SpxR and CpsR, work in concert to regulate lung infection, lung-to-blood transition and sepsis through a distal *cis*-acting DNA element we have named the *37-CE*.

## Results

### Identification of *cps* promoter and *37-CE*-interacting transcription factors

**Fig 1:**
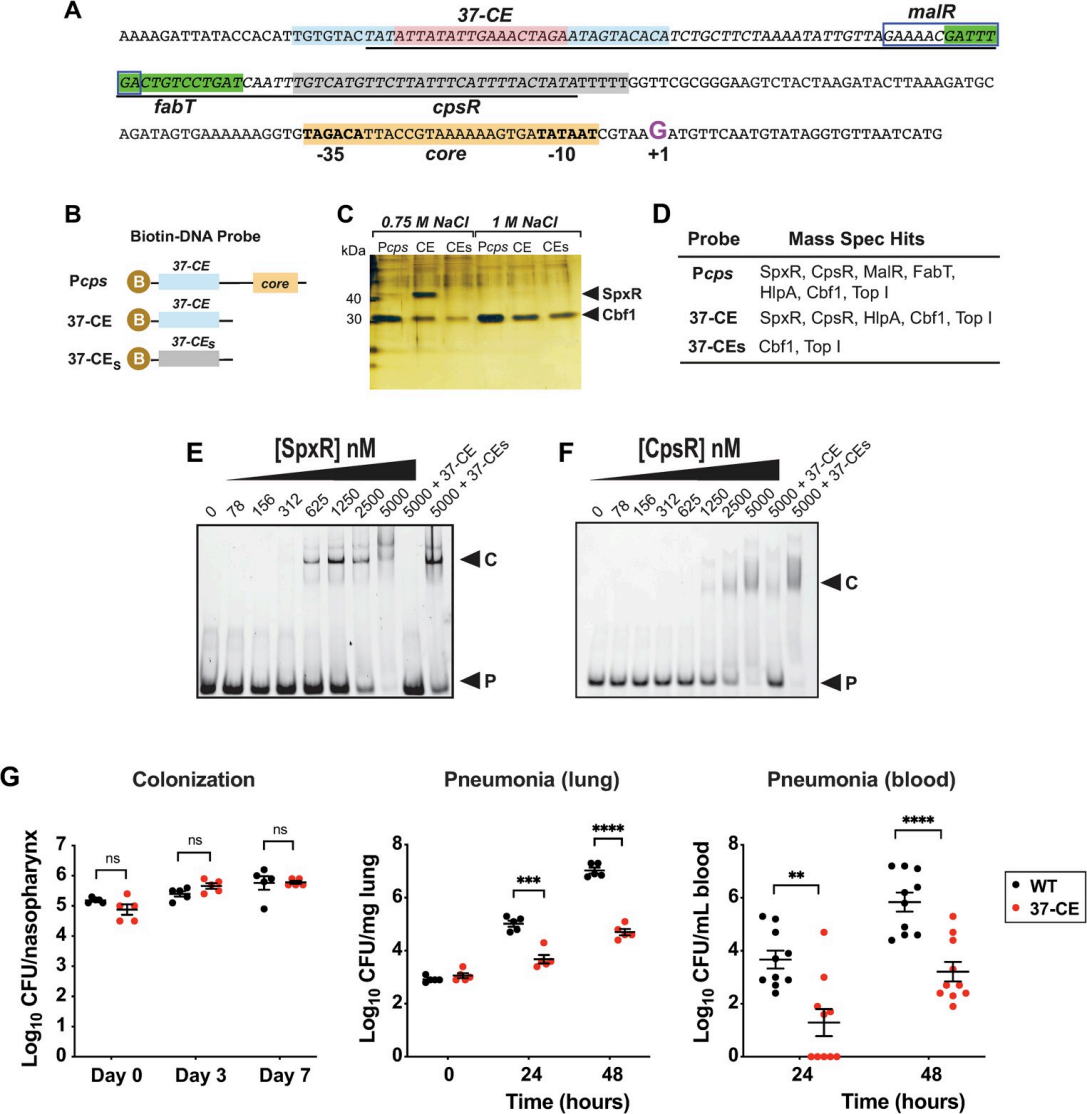
Identification and characterization of the *37-CE*. (**A**) Annotated P_*cps*_ sequence. The *37-CE* is highlighted; the 10 bp perfect inverted repeat sequence is light blue while the 17 bp spacer region is pink. Proposed FabT and CpsR binding sites as identified in refs. (27) and (19, 26) are in green and grey, respectively. The proposed MalR binding site according to refs. (24, 25) is boxed. Core promoter elements are highlighted in orange. The Repeat Unit of Pneumococcus (RUP) sequence (34, 35), is underlined and the +1 transcriptional start site (G) is in purple. (**B**) Biotin (B) labelled DNA probes used to pull-down P_*cps*_ interacting partners. P_*cps*_ = full *cps* promoter; *37-CE* = 37 bp *cis* element; *37-CEs* = scrambled *37-CE* sequence. (**C**) Silver-stained SDS-PAGE of biotin-conjugated DNA probe high salt eluates after bacterial lysate was bound and washed. SpxR and Cbf1 are indicated by the arrows. (**D**) Table of transcription factors identified using Mass Spectrometry (MS) by the three probes. (**E**) Representative EMSA of SpxR and (**F**) CpsR interaction with *37-CE* double-stranded DNA probe. Excess unlabeled probe (+*37-CE*) or scrambled probe (+*37-CEs*) are used as controls. EMSAs were performed three times. (**G**) Murine infection studies. For colonization (left), mice were infected with 5x10^5 CFU and CFUs were determined on day 3 (5 mice/group) and day 7 (5 mice/group). For pneumonia, mice were infected with 5x10^6 CFU and CFUs were determined in lung homogenates (middle; 5 mice/group) and blood (right; 10 mice/group) at 24 and 48 hours post-infection. Individual data points, the mean and SEM are plotted. Statistical differences were determined using an unpaired t-test. Symbols: ns = not significant ***p≤0.001, ****p≤0.0001.

To identify potential *cps* promoter-interacting transcription factors (TFs), we used a method described by Jutras *et al*. [21]. Biotinylated DNA probes of the *cps* regulatory region encompassing +15 to -230 (P_*cps*_), and a 37 bp region (*37-CE*) identified as containing a perfect inverted repeat sequence at the -172 position (**Fig 1A**) were used to isolate TFs from pneumococcal lysates, along with a scrambled *37-CE* as a control (*37-CE*_*s*_; **Fig 1B**). Bound protein was eluted with increasing salt concentrations before identification by silver staining and mass spectrometry (MS) analysis.

The silver-stained gel indicated an obvious protein close to 47 kD present in both P_*cps*_ and *37-CE* samples, but not the *37-CE*_*s*_ scrambled control (**Fig 1C and 1D**). This band was determined by MS to be SpxR, a multidomain TF required for murine lung infection [22]. Both *cmp*-binding protein (Cbf-1, [23]) and Topoisomerase I (Top I) were identified in the three elution conditions and the control (**Fig 1C and 1D**), and were therefore not deemed to be specific. Examination of the whole 750 mM P_*cps*_ eluate revealed a total of 4 DNA-interacting proteins with more than one identified peptide and a significant Mascot score over 100 (**Figs 1D, S1A and S1B)**: SpxR (SPD_0969), maltose-regulated MalR (SPD_1938) [24,25], putatively glucose-regulated CpsR (SPD_0064; also called AgaR) [19,26], and the histone-like DNA supercoiling protein, HlpA (SPD_0997). The fatty acid regulatory TF FabT (SPD_0379) [27,28] was also identified by a single peptide, which has been confirmed by others to interact with the *cps* regulatory region [16,19]. Using the same stringent criteria, a comparative analysis of the *37-CE* and *37-CE*_*s*_ 750 mM eluates revealed that SpxR, CpsR and HlpA were all potential, specific *37-CE*-interacting TFs.

### Both SpxR and CpsR specifically bind to the *37-CE* sequence

To determine if SpxR and CpsR specifically interacted with the *37-CE* sequence, Electrophoretic Mobility Shift Assays (EMSAs) were used. Recombinant SpxR and CpsR were purified to near homogeneity (**S1C Fig**) and increasing amounts were added to a fixed amount of fluorescently-labelled *37-CE* DNA probe (**Fig 1E and 1F**). SpxR began to shift with as little as 312 nM protein and CpsR with 625 nM. Importantly, binding specificity was demonstrated by TF interactions being competed with the same unlabeled probe, but not scrambled probe.

### The *37-CE* is required for pneumococcal pneumonia

SpxR and CpsR have been shown to influence the expression of numerous genes and consequently, deletion of SpxR and/or CpsR could result in pleiotropic effects [19,26]. We therefore aimed to evaluate the contribution of SpxR and CpsR specific to *cps* regulation *in vivo* through disruption of their interaction with the *37-CE*. To accomplish this, we constructed an isogenic deletion of the *37-CE* sequence (Δ*37-CE*) in *S*. *pneumoniae* strain D39 (serotype 2) [11] and performed murine colonization and pneumonia models as per ref. (29).

Results indicated no appreciable difference in colony forming units (CFUs) between WT and the Δ*37-CE* mutant after 7 days of colonization (**Fig 1G**). In contrast, when a pneumonia model of infection was implemented, a greater than two log decrease in CFUs of the Δ*37-CE* mutant were recovered from the lungs and blood of mice compared to WT at both 24 and 48 hours post-inoculation (**Fig 1G**). These results show that the *37-CE* is not required for a week-long colonization of a naïve mouse, but is important for pneumonia, and may be required for the lung-to-blood transition.

### Identification of SpxR and CpsR binding sites

To evaluate the individual contributions of SpxR and CpsR regulation through the *37-CE*, we needed to identify the minimal nucleotides required for binding of each TF. This was achieved by first creating a series of truncated *37-CE* oligos and performing EMSAs with either purified SpxR DNA-binding domain (SpxR_DBD_; monomeric) or CpsR (dimeric) (**S1C, S1D and S2 Figs**). A detailed description of this process is described in **S2 Fig.**

**Fig 2:**
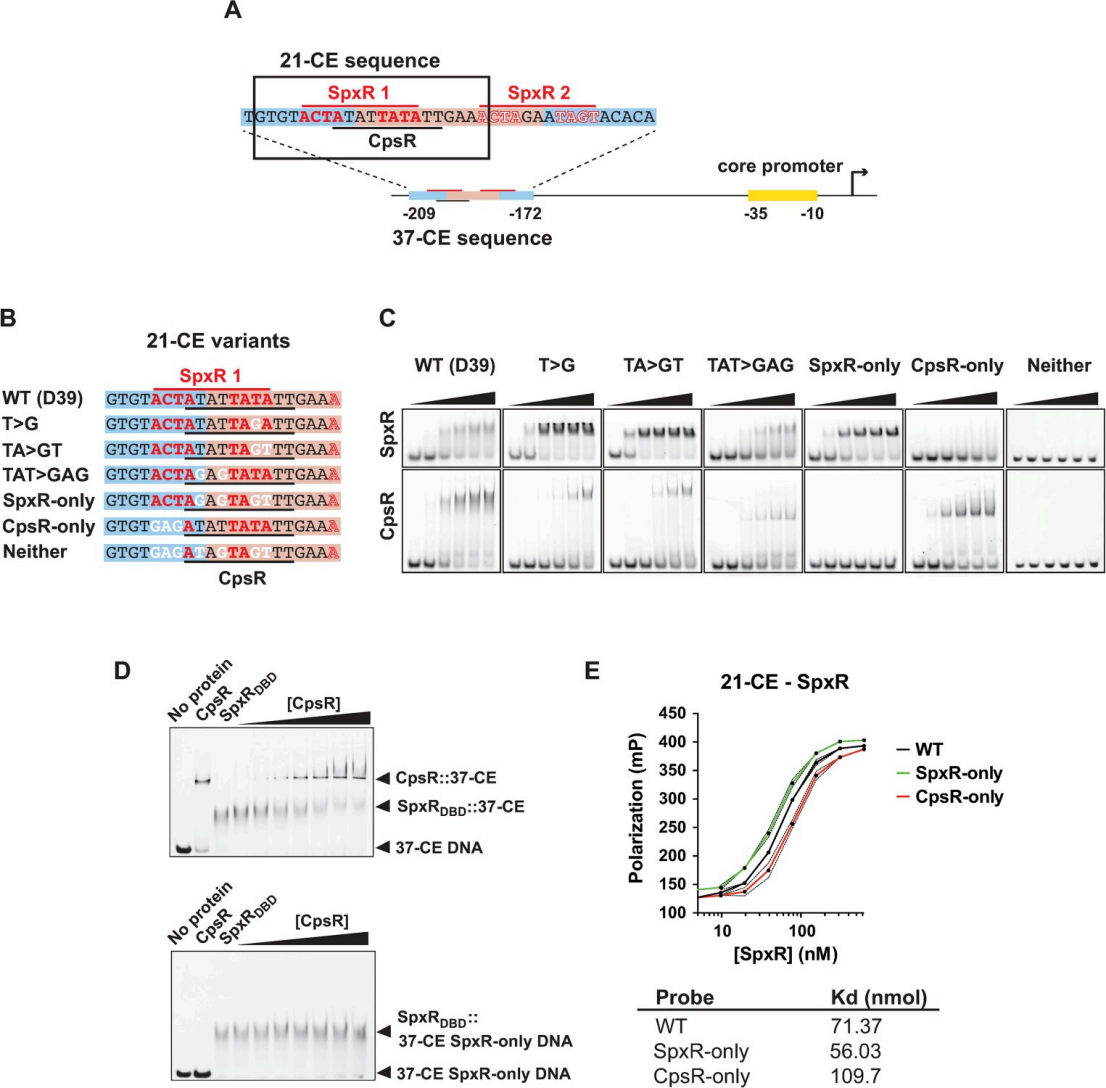
SpxR and CpsR binding to the *37-CE*. (**A**) Schematic diagram of the *cps* promoter and *21*/*37-CE* sequences. The *21-CE* sequence is boxed. (**B**) Oligos used to determine specific nucleotides required for SpxR and CpsR binding. Red line and red residues: proposed *SpxR1* inverted repeat binding site and minimal required nucleotides for interaction. Black line: proposed refined CpsR binding site (this work). White labelled nucleotides were mutated. (**C**) Representative EMSAs of *21-CE* oligos depicted in *B*. (**D**) (Above) representative EMSA showing that CpsR competes for SpxR_DBD_ binding to the *37-CE* oligo. (Below) representative control EMSA showing that CpsR is unable to compete for SpxR_DBD_ binding in the absence of its binding motif. EMSAs were performed three times. (**E**) Fluorescence polarization of WT, *SpxR-only* and *CpsR-only 21-CE* DNA variants with full-lenfth SpxR protein. Polarization is expressed in millipees (mP). The mean of three independent experiments is plotted. Error bars represent standard deviation and are indicated by dashed lines. Average equilibrium dissociation constants (Kds) are given below.

SpxR_DBD_ was found to interact with two 11-mer inverted repeat sequences, one imperfect (*spxR1*) and one perfect (*spxR2*), within the *37-CE* (**Fig 2A**). CpsR was found to interact with a 10-mer direct repeat that resembled a previous report’s motif [19,25] whose sequence partially overlapped with the imperfect *spxR1* binding site (**Fig 2A**; refs 19 and 26). Next, we identified the minimal nucleotides required for interaction with a refined 21-mer sequence (*21-CE*) consisting of the *spxR1* and CpsR sites. Using a series of point mutations (*i*.*e*. T>G, TA>GT and TAT>GAG; **Fig 2B**) we were able to mutate four nucleotides to abolish CpsR interaction while retaining SpxR_DBD_ interaction, but with greater affinity (*SpxR-only*; **Fig 2C**), and conversely, mutate three different nucleotides to abolish SpxR_DBD_ interaction while retaining CpsR interaction, but with greater affinity (*CpsR-only*; **Fig 2C**). Combining both sets of mutations prevented both SpxR and CpsR from interacting (*Neither*; **Fig 2C**). Knowing that the SpxR and CpsR binding sequences were overlapping, we determined if the two TFs could compete for interaction with the *21-CE*. Titration of CpsR into a SpxR_DBD_ /*21-CE* DNA reaction mixture indeed resulted in competitive occlusion of SpxR_DBD_ interaction (**Fig 2D,** *above*), but not if the CpsR binding site was removed through mutation (**Fig 2D**, *below*), confirming that SpxR and CpsR can compete for interaction with overlapping binding sites within the *21-CE* sequence.

According to size exclusion chromatography (SEC) experiments, SpxR forms a tetramer in solution whereas CpsR forms a dimer (**S1D Fig**). We were unable to produce consistent EMSAs with full-length SpxR protein and the *21-CE*, which we deemed likely due to the large size of the SpxR oligomer. Therefore, we used an in-solution binding method, fluorescence polarization (FP), to further interrogate SpxR’s interaction with the *21-CE*. Using FP, we calculated a Kd of 71 nM (**Fig 2E**). In line with EMSAs (**Fig 2C**), SpxR interacted with *SpxR-only* with higher affinity (56 nM), and with lower affinity to *CpsR-only* (104 nM) (**Fig 2E**). We were unable to obtain saturating, sigmoidal binding curves with CpsR protein, likely because of its relatively small size in solution (65.72 kDa as a dimer) compared to the size of the 6-FAM-labeled DNA (**S1C, S1D and S2C, S2D Figs**).

### SpxR and CpsR repress capsule expression *in vitro*

**Fig 3:**
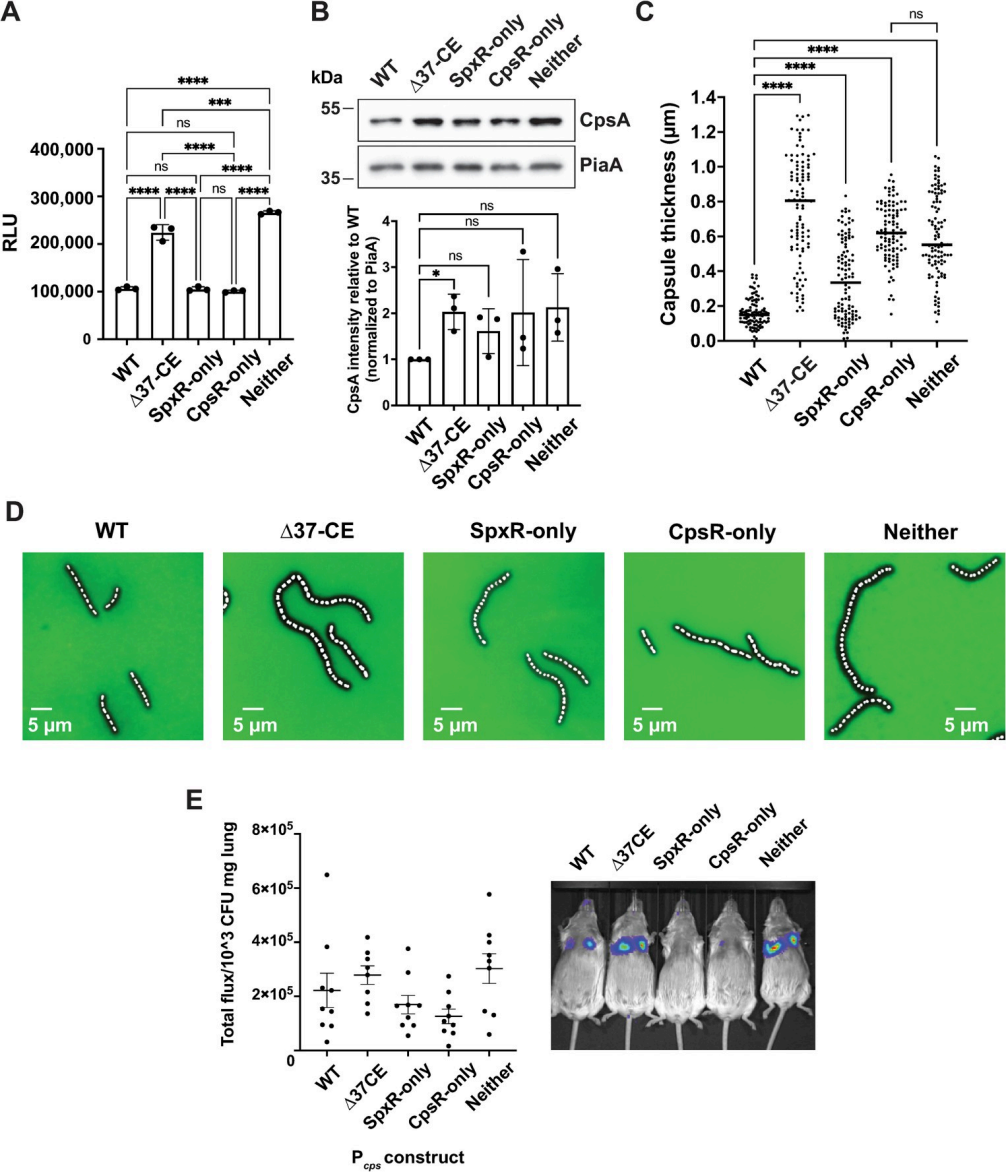
*In vitro* regulation of the capsule by SpxR and CpsR through the *37-CE* and *in vivo* imaging studies. (**A**) Luciferase reporter assay in Δ*37-CE*, and strains where only SpxR (*SpxR-only*), CpsR (*CpsR-only*), or neither SpxR nor CpsR can bind (*Neither*) the *37-CE* sequence. Individual data points, the mean, and SEM are plotted from three biological replicates. RLU; relative light units. (**B**) Western blot analysis of CpsA protein. A representative blot of three independent experiments is shown with quantification below. (**C**) Quantification of capsule content using dextran exclusion assay. Individual cells from five biological replicates, the mean, and SEM are plotted. (**D**) Representative images of *C* showing a fluorescence and phase contrast microscopy overlay. The dark area around the cell represents the capsule. (**E**) (Left) *In vivo* imaging quantification (total luminescence over lung normalized to CFU/mg of lung tissue) of P_*cps*_::*CBRluc* reporters during a pneumonia model of infection. (Right) Example images of one set of mice. Statistical differences in A and C were determined using a one-way ANOVA with Tukey’s multiple comparisons test. Statistical differences in B were determined using a one sample t test and Wilcoxon test. ns = not significant, *** p≤0.001, **** p≤0.0001.

Having identified mutations that allowed us to assess the individual contributions of SpxR and CpsR to *cps* regulation, we then investigated their influence on promoter activation, translation, and capsule production. For P_*cps*_ activation we constructed a new integrative plasmid, pPP3, that enabled us to monitor promoter activity using a red-emitting click beetle luciferase (P_*cps*_::*CBRluc*) reporter (**S3A and S3B Fig**). Deletion of the *37-CE* resulted in an approximate 2-fold increase in P_*cps*_ activity. This was phenocopied by the *Neither* construct, suggesting that the *37-CE* is required for repression of P_*cps*_ activation (**Fig 3A**). Interestingly, when only one or the other TF is able to bind with greater affinity (*SpxR-only* and *CpsR-only*), WT levels of P_*cps*_ activation were observed, consistent with one TF being able to compensate for the other in its absence under these conditions.

To assess the effect of SpxR and CpsR on *cps* translation and capsule production, we created isogenic *21-CE* mutant strains harboring the *SpxR-only*, *CpsR-only*, and *Neither* mutations (**Fig 2B**) and then measured CpsA protein levels (the first gene of the *cps* operon) by western blot, and capsule thickness using the dextran-exclusion method [30]. Differences in CpsA protein levels between strains largely paralleled our luciferase reporter data, further enforcing the hypothesis that CpsR and SpxR repress *cps* transcription through the *37-CE* (**Fig 3B**). Quantification of capsule production with microscopy also paralleled that of P_*cps*_ activation and translation data (**Fig 3C and 3D**). One exception was the *CpsR-only* strain, where average capsule thickness was less than that of the Δ*37-CE* strain, but not as repressed as seen in our luciferase reporter data or CpsA protein levels.

### *In vivo* imaging of P_cps_ activity

Having determined the role of SpxR and CpsR in *cps* regulation *in vitro*, we wanted to measure P_*cps*_ activity during a pneumonia model of infection using the luciferase reporter strains in **Fig 3A**. Light was measured 4 hours post-infection using an IVIS imager and the signal was normalized to CFUs per mg of lung tissue. P_*cps*_ activity during the pneumonia model mirrored our *in vitro* data; deletion of the *37-CE* (Δ*37-CE*) caused an increase in P_*cps*_ activity, the *SpxR-* and *CpsR-only* constructs mirrored WT, and the *Neither* construct had higher activity than the Δ*37-CE* construct (**Fig 3E**). These data are consistent with the hypothesis that SpxR and CpsR play compensatory roles during lung infection to repress capsule production through the *37-CE*.

### SpxR and CpsR control pneumococcal infection through the *37-CE*

**Fig 4:**
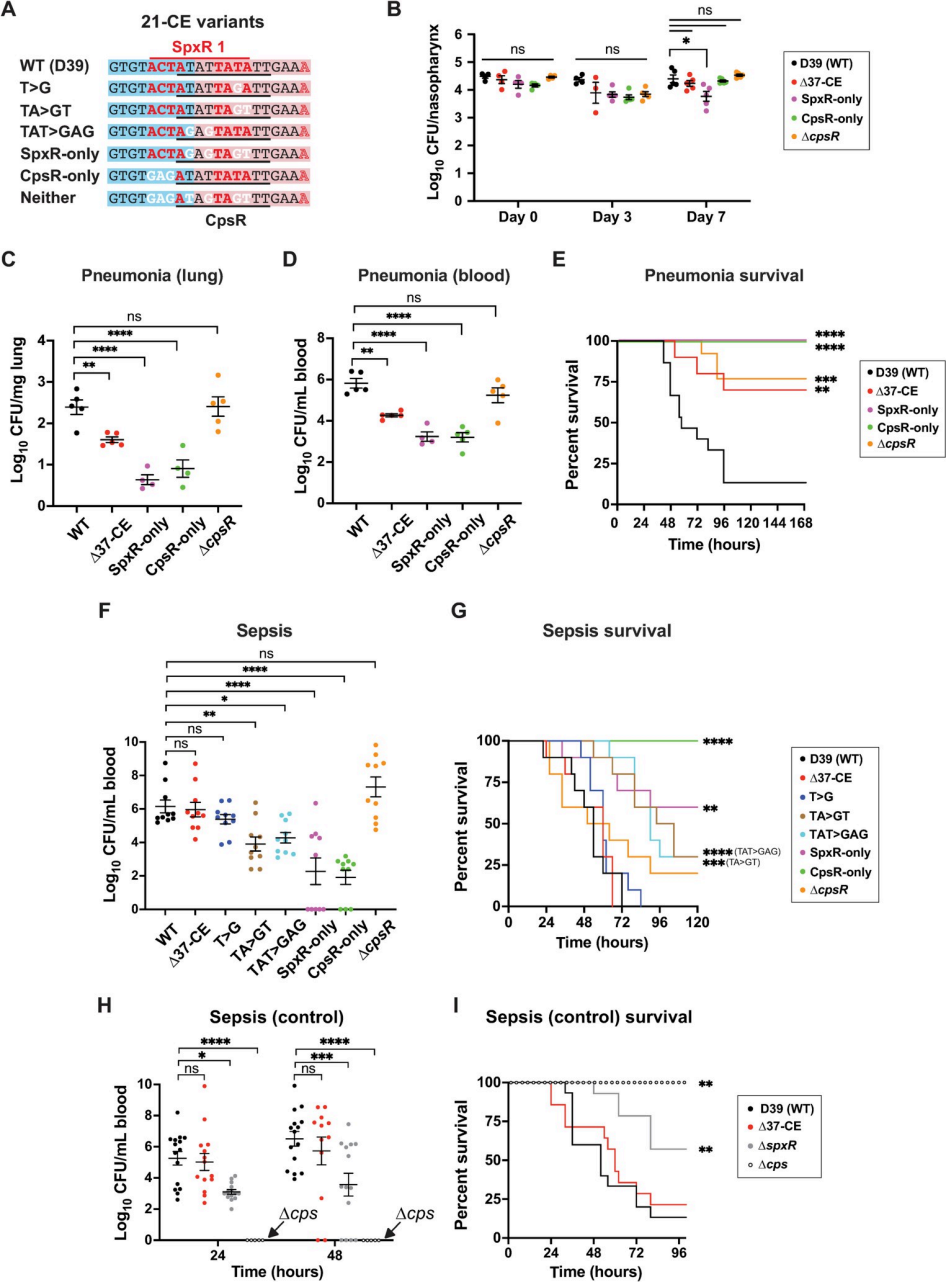
Infection studies. (**A**) Representative *21-CE* variants used in infections. (**B**) Colonization model. Mice were infected intranasally with 5x10^5 CFUs of *37/21-CE* variants. CFUs were enumerated in nasopharyngeal washes on day 0, 3 and 7. (**C, D & E**) Pneumonia model. Mice were infected intranasally with 5x10^6 CFU. CFUs in lungs (*C)* and blood (*D*) were enumerated at 24 hours post-infection. Mouse survival is plotted in (*E*). (**F & G**) Sepsis model. Mice were infected via the dorsal tail vein with 2x10^6 CFU. Blood was withdrawn from the tail vein at 24 and 48 hours for enumeration of CFUs (*F*). Survival of mice from *F* are plotted in (*G*). (**H & I**) A sepsis model as performed in *F* with Δ*spxR* and Δ*cps* strains. Survival of mice from *H* are plotted in (*I*). *Statistics*: For *B*, a two-way ANOVA was used with Dunnett’s multiple comparison test to determine differences between strains on the same day, and Tukey’s multiple comparison test to determine differences between the same strain on different days. For *C* and *D* a one-way ANOVA with Dunnett’s multiple comparison was used. For *F* and *H* a two-way ANOVA with Tukey’s multiple comparison test was used. Statistical differences in survival in *E*, *G*, and *I* were determined using the Kaplan-Meier and Log-rank tests for multiple comparisons. ns = not significant; * p≤0.05; ** p≤0.01; *** p≤0.001; **** p≤0.0001. Individual data points, the mean and SEM are plotted.

Having established the requirement for the *37-CE* in a pneumonia model of infection (**Fig 1G**), we next sought to evaluate the individual contributions of SpxR and CpsR to *cps* regulation during colonization, pneumonia and sepsis using the aforementioned *21-CE* isogenic mutants (**Fig 4A**). In some experiments, D39 Δ*spxR*, Δ*cpsR* and Δ*cps* deletion mutants were also incorporated. Importantly, the *37/21-CE* variants exhibited no growth defects when cultured *in vitro* (**S4 Fig**).

Our colonization model revealed no significant differences in CFUs recovered between WT and *21-CE* variants, except for a minor decrease in the *SpxR-only* strain (**Fig 4B**). However, in the pneumonia model, we observed an approximate 2 log decrease in CFUs of both the *SpxR-only* and *CpsR-only* variants recovered from the lungs (**Fig 4C**, p≤0.0001) and blood (representative of lung-to-blood transition and replication in the blood; **Fig 4D**, p≤0.0001) compared to WT. Coinciding with these observations, *SpxR-only* and *CpsR-only* variants were avirulent (**Fig 4E**, p≤0.0001), most likely as a consequence of *cps* dysregulation causing increased clearance in the lungs and blood. Despite no significant difference in CFUs between Δ*cpsR* and WT (**Fig 4C and 4D**), Δ*cpsR*-infected mice maintained a 75% survival rate (**Fig 4E**, p≤0.01), suggesting that after initial rounds of replication the Δ*cpsR* mutant begins to be cleared.

Pneumococcal pneumonia mortality rates are largely driven by its ability to both transition to and survive within the blood following lower airway infection [4]. Although we observed significant decreases in CFUs isolated from blood collected during our pneumonia model (**Fig 4D**, p≤0.0001), it was not clear whether this decrease was due to a defect in their ability to transition into the blood, or an inability to survive in the blood; an environment where the capsule is critical for avoiding phagocytosis and antigen recognition [5,31]. To investigate further, we assessed the ability of *21-CE* variants (shown in **Fig 4A**) to survive in the blood using a sepsis model, the results from which the lung-to-blood transition would not have to be considered. Owing to its importance in avoidance of phagocytosis [9], the capsule is essential for survival in the blood, therefore differences observed in survival of the *37/21-CE* variants are most likely due to differences in capsule expression in this model. Results shown in **Fig 4F** clearly demonstrate that deletion of the *37-CE* sequence did not result in a significant decrease in CFUs during sepsis, suggesting that capsule expression in this strain is comparable to WT. However, both the *SpxR-only* and *CpsR-only* strains were severely attenuated, exhibiting an almost 4-log decrease in CFUs (**Fig 4F**, p≤0.0001). These strains were either highly attenuated in virulence (*SpxR-only*, p≤0.01) or completely avirulent (*CpsR-only*, p≤0.0001), suggesting that these mutations cause a decrease in capsule expression in the blood compared to WT. The *TA>GT* and *TAT>GAG* variants which, with respect to SpxR and CpsR’s ability to interact with the *21-CE* are intermediates of the *SpxR-only* and *CpsR-only* variants (**Fig 2C**), displayed near 2-log decreases in CFUs (**Fig 4F**, p≤0.01 and p≤0.05 respectively) and were mildly attenuated in virulence (**Fig 4G**, p≤0.001 and p≤0.0001 respectively). Together, these results are again consistent with a decrease in capsule expression in the blood compared to WT. The *T>G* variant, which also has intermediate changes in SpxR and CpsR’s ability to interact with the *21-CE* (**Fig 2C**), was not significantly different from WT in the sepsis model. This could suggest that there is another factor that is influenced by the mutations in the *21-CE* that prevents derepression, such as the HlpA protein, which was identified as being capable of interacting with the *37-CE* and *cps* promoter in our initial pulldown (**Fig 1D**). Similar to the pneumonia model, deletion of *cpsR* (Δ*cpsR*) did not have an effect on sepsis model CFUs when compared to WT (compare **Fig 4F** and **4D**), unlike deletion of *spxR* (about a 2 log reduction; **Fig 4H**, p≤0.05). Paralleling a previous report [19], mice infected with the Δ*cpsR* mutant trended towards a slightly higher survival rate than WT (20% vs 0%; **Fig 4G**), though this difference was not statistically significant. In contrast, deletion of *spxR* led to an intermediate survival phenotype that was statistically significant (57%; **Fig 4I**, p≤0.01). The differences observed between the *spxR* and *cpsR* deletion mutants in this model suggest that unlike CpsR, SpxR regulates additional genes in the blood that are required for pneumococcal survival.

Together, these data demonstrate that in strain D39 (serotype 2), SpxR and CpsR work together through the *37-CE* to repress capsule expression during pneumonia and lung-to-blood transition (**Fig 4C–4E**), whereas during sepsis—where we expect *cps* transcription to increase [32]—SpxR and CpsR derepress capsule expression, as evidenced by the *37-CE* not being required in this environment (*Δ37-CE* strain: **Fig 4F–4H**). The repressive role of SpxR and CpsR through the *37-CE* is confirmed by the *SpxR-only* and *CpsR-only* mutations; the increased affinity of binding overrides signaling that would otherwise cause derepression in the blood, leading to inappropriate repression of capsule expression and therefore, an avirulent phenotype (**Fig 4E**).

### SpxR quaternary structure

**Fig 5:**
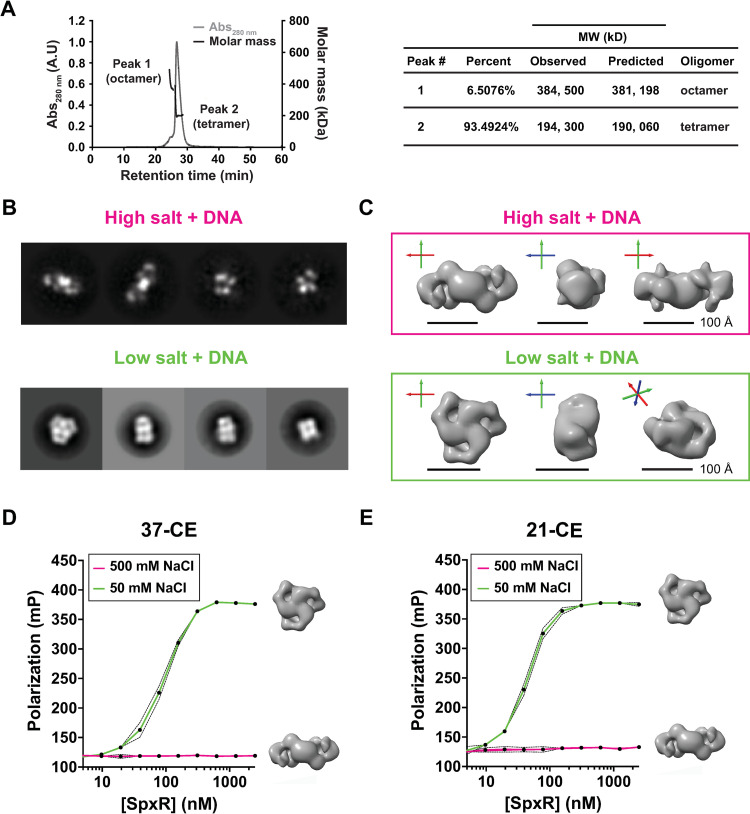
Quaternary structure of SpxR. (**A**) SEC-MALS analysis (left) and numeric mass data (right) of SpxR in high salt (500 mM NaCl). A major tetramer peak and a minor octamer peak were observed. (**B**) EM negative stain 2D class averages of SpxR with DNA in both high and low salt. (**C**) Low resolution 3D models for SpxR with *37-CE* DNA in high salt (tetramer) and low salt (trimer “wheel”). The diagram shows three views of the complexes obtained from the EM negative stain data. The long axis of the tetramer complex (high salt) is approximately 200 Å long, whereas the diameter of the trimeric (low salt) wheel is approximately 135 Å. In the low salt complex there is a visible opening formed by the domains, which could be where the double stranded DNA resides. For reference, the three Cartesian axes are shown (X red, Y green, Z blue) on each picture. (**D**) Fluorescence polarization of SpxR titrated into either *37-CE* DNA or (**E**) *21-CE* DNA, in either low or high salt buffer (Tris pH 7.0 with 50 mM NaCl or 500 mM NaCl, respectively). Polarization is expressed in millipolarization units (mPs). The mean of three independent experiments is plotted. Error bars represent standard deviation and are indicated by dashed lines.

To better understand the mechanism by which SpxR interacts with the *37-CE* and thereby regulates capsule expression, we first performed SEC coupled to Multi-Angle Light Scattering (SEC-MALS), which gives a precise molecular weight estimation of molecules in solution. In the absence of DNA, recombinant SpxR requires supraphysiological concentrations of salt to prevent precipitation, therefore SEC-MALS could only be performed under high salt (500 mM) conditions. SEC-MALS paralleled our SEC results from **S1D Fig**, where it exhibited a mostly tetrameric oligomer state. However, an octameric species was also present (**Fig 5A**). To further characterize the quaternary structure of SpxR we exploited negative stain electron microscopy (EM). SpxR was examined in the presence of *37-CE* DNA in both a low salt (50 mM NaCl) and a high salt (500 mM NaCl) buffer. The EM data showed clear two-dimensional (2D) class averages (**Fig 5B**) and it was possible to obtain low-resolution three-dimensional (3D) reconstructions of the molecule under both conditions (**Fig 5C**). Under high salt conditions, where SpxR does not bind DNA as measured by FP (**Fig 5D and 5E**), we observed an ellipsoidal shaped particle depicting C2 symmetry and approximate dimensions 64 Å x 86 Å x 196 Å, putatively the tetrameric species from the SEC MALS experiments (which was also performed under high salt conditions; **Fig 5C**). The low salt sample, which binds both *37-CE* and *21-CE* DNA (**Fig 5D and 5E**), showed a strikingly different conformation, exhibiting C3 symmetry with a smaller and more compact size that resembled a short trimeric cylindric “wheel”, with approximate dimensions 135 Å in diameter and 88 Å in height. Taken together, these results tell us that SpxR is likely to associate in a trimeric configuration in the presence of DNA under more physiological (*i*.*e*. low salt) conditions.

### IR conservation in pneumococcal serotypes

**Fig 6:**
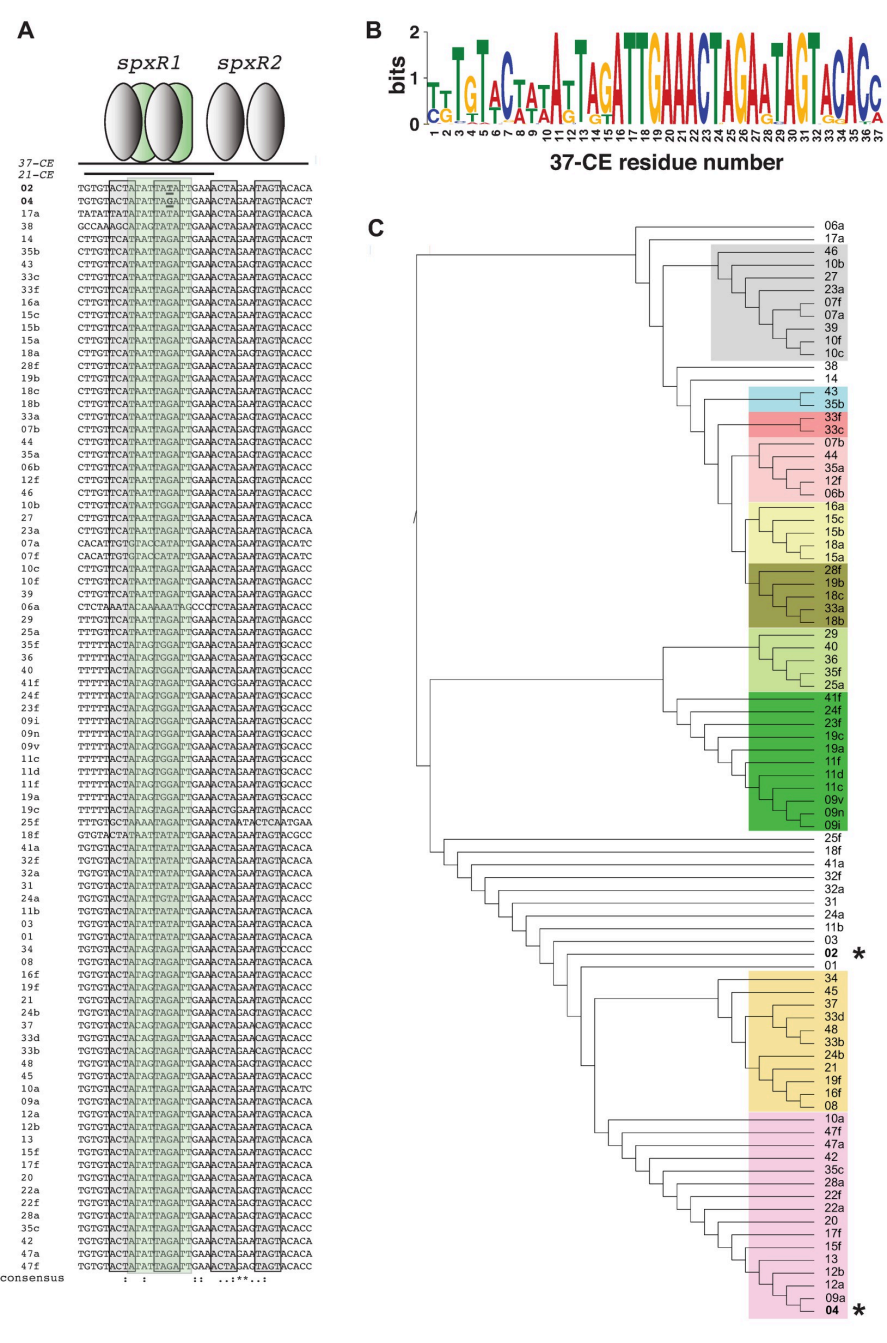
Analysis of the *37-CE* / *21-CE* sequence in different pneumococcal serotypes. (**A**) Sequences were obtained from the EMBL database (33) and aligned using Clustal Omega (53). The four proposed SpxR ACTA sequences within the *37-CE* are highlighted in grey. Proposed CpsR sequences are highlighted in green. D39 (serotype 2) and TIGR4 (serotype 4) are labelled in bold and found at the top of the alignment. (**B**) Meta-MEME (54) generated a motif using the sequences in *A* displayed in Hidden Markov Model (HMM) Logo format. (**C**) Phylogenetic tree generated using Clustal Omega from the *37-CE* alignment shown in *A* displayed using iTOL (55). *37-CE* clades are highlighted in different colors. Asterisks indicate serotype 2 (D39) and serotype 4 (TIGR4).

Thus far, we have shown that SpxR and CpsR work though the *37-CE* to regulate pneumococcal infection, lung-to-blood transition and sepsis. However, these findings begged the question as to how universal this mechanism was, given the 100+ known serotypes [12] and strain variances [33]. Analysis of the *37-CE* sequence from 87 serotypes revealed that the 3’ end of the *37-CE* that contains the *spxR2* binding site lies within the conserved Repeat Unit of Pneumococcus (RUP) sequence [34,35], and conversely that the 5’ end (*i*.*e*. the *21-CE* portion of the sequence that contains the *spxR1* binding site; see **Fig 2A**), which is partially outside of the RUP, is more variable between serotypes (**Figs 1A, 6A and 6B**). When a phylogenetic tree of the *37-CE* sequences was generated, specific clusters of sequences could be discerned, suggesting regulatory conservation between subsets of serotypes (**Fig 6C**).

When combined with our data demonstrating the sensitivity of this region to fine-tuning SpxR/CpsR binding, capsule regulation and pneumococcal virulence, this observation suggests that the *37-CE* could serve as an important focal point of inter- and intra-serotype specific invasive variation through SpxR and CpsR interactions.

## Discussion

A longstanding challenge in pneumococcal biology has been to understand how this debilitating pathogen regulates its major virulence factor, the capsule, to achieve successful colonization and downstream infections. In this work, we reveal that the transcriptional mechanism behind *cps* regulation is mediated by two TFs—SpxR and CpsR—through the *37-CE*, a previously unknown, *cis*-acting, distal regulatory element. Both *in vitro* and *in vivo* data point to a mechanism whereby SpxR and CpsR work in concert to repress *cps* transcription within the lower airways through overlapping binding motifs, and then derepress transcription in the blood. Indeed, when either i) the *37-CE* is deleted (Δ*37-CE*), ii) one TF’s ability to bind the *37/21-CE* is abolished, increasing the affinity of the other (*SpxR-only* and *CpsR-only*), or iii) the two TFs are independently deleted (Δ*spxR* and Δ*cpsR*), orders of magnitude reductions in CFUs and/or a concomitant increase in host survival were observed during pneumonia. However, during sepsis, deletion of the *37-CE* did not affect bacterial replication or mouse survival (**Fig 4F–4I**). Assuming that SpxR and CpsR are the dominant repressors of *cps* transcription, as indicated by this work, we would expect environmental/cellular signals in the blood to lead to derepression by these TFs, a hypothesis that is consistent with the *37-CE* being dispensable in this environment. Interestingly, the *SpxR-only* and *CpsR-only* variants led to a profound loss of infectivity during sepsis (**Fig 4F–4I**). This phenomenon could be attributed to the aforementioned changes in binding affinity associated with disrupting either TF, which may then enable them to overcome signal-mediated derepression during sepsis, ultimately resulting in the inappropriate repression of *cps* transcription and therefore decreased bacterial survival. One limitation of this study is that the initial pulldown was performed using lysates of pneumococci grown under standard growth conditions in rich medium. Therefore, it is possible that there are TFs that are only expressed/only interact with the *cps* promoter *in vivo* that were not identified. It follows that derepression of *cps* transcription in the blood may occur as a consequence of SpxR and CpsR being displaced by another as of yet unidentified TF that binds with high affinity in the blood, instead of/alongside ligand-induced signaling events that decrease SpxR and CpsR’s affinity for the *37-CE*.

The pneumococcus is a facultative anaerobe lacking a complete TCA cycle [36]; therefore, both the amount of molecular oxygen and sugars available to metabolic pathways are likely key factors in determining when changes in capsule expression can be afforded. In line with this thinking, the capsule is diminished in the oxygen-rich nasopharynx and lower airways where host glycoconjugates are the predominant sugar source [37] and, conversely, expanded in the relatively anaerobic, glucose-rich environment of the blood, where most molecular oxygen is bound by hemoglobin (~98.5%) [38]. We hypothesize that SpxR coordinates capsule expression with central metabolism. In support, the paper that originally described SpxR revealed that it strongly influences expression of the pneumococcal pyruvate oxidase, SpxB, an oxygen-dependent central metabolic enzyme whose activity has been shown to drive changes in capsule expression [22,39–42].

**Fig 7:**
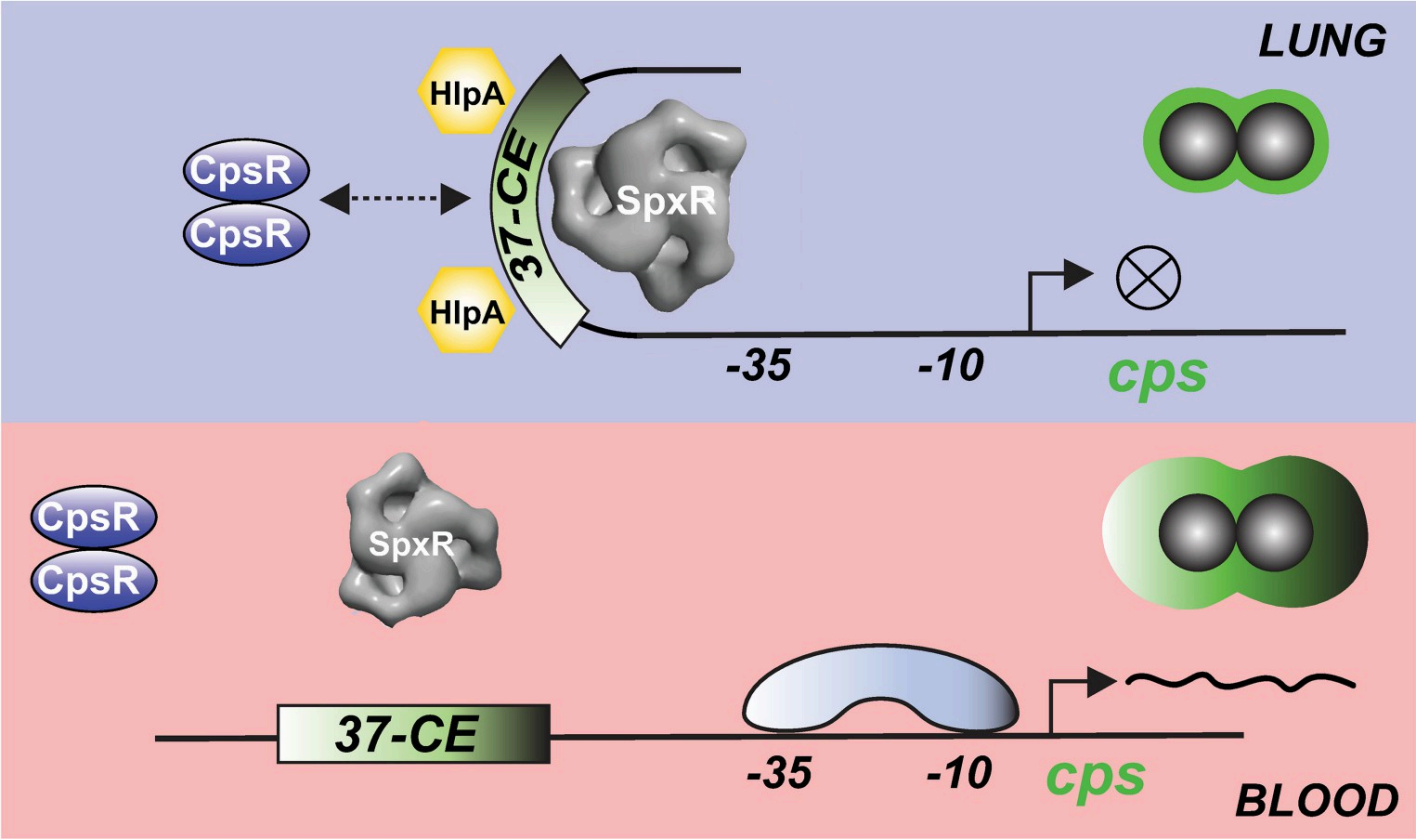
Schematic representation of the proposed mechanism of *cps* regulation during pneumococcal infection. SpxR and CpsR respond to unknown ligands in the airways whose presence or absence could influence their interaction with the *37-CE*. SpxR binds as a trimer and CpsR as a dimer. Interaction is associated with the nucleoid-like protein HlpA, which assists in SpxR and/or CpsR repression by bending the DNA to occlude RNAP promoter access. After lung to blood transition, we hypothesize that changes in ligand concentrations result in the de-repression of *cps* operon transcription by alleviating transcription factor binding and allowing for RNAP recruitment. The capsule machinery (*cps* operon) is then increased substantially during sepsis to avoid phagocytosis. The importance of SpxR and CpsR in regulating *cps* control might be serotype-specific depending on the precise composition of the *37-CE* sequence.

Previous *en masse* transcript studies comparing WT D39 and a Δ*spxR* deletion mutant did not identify differentially regulated *cps* locus genes, though this discrepancy could be explained by the use of an R6 genome array in these experiments—a serotype 2-derived strain that lacks a capsule [11]. The authors suggest that SpxR could be positively regulating SpxB in the airways [22]. When combined with our data here, a model of SpxR regulation of pneumococcal infection emerges where SpxR drives SpxB activity in the oxygen-rich airways, while repressing *cps* transcription, then derepresses *cps* transcription in the blood following a shift in metabolism caused, in part, by a reduction in SpxB activity (a result of the near absence of molecular oxygen). Although this model is likely oversimplified considering the array of TFs that regulate the *cps* locus (**Fig 1**), SpxR, CpsR and the *37-CE* clearly play critical tissue-specific roles in capsule regulation during infection. **Fig 7** summarizes our results.

One important missing piece of information in our work is precisely what signal(s) SpxR and CpsR respond to. The paper that originally described CpsR identified TFs that interact with the *cps* regulatory region using a similar approach, however their biotinylated *cps* DNA sequence did not include the *37-CE*, which could explain why they did not also identify SpxR as a *cps* regulatory TF [19]. Their work, which focuses on a different, more proximal CpsR binding site (**Fig 1A**), suggested that glucose addition inhibits CpsR from binding to DNA, which along with being the predominant sugar source in the blood makes glucose a strong candidate for a prospective ligand [19]. Keeping this in mind, we tested if the addition of glucose and several other potential ligands at comparable ratios to ref. 19 (1000X, 5 mM sugar to 5 μM protein) could influence CpsR binding to the *21-CE* and full *cps* regulatory region with no success (**S5A and S5B Fig**). This discrepancy could be due to us using a different binding site, or something else. It will be interesting to further examine the responsiveness of CpsR to glucose in the context of what else has been discovered here. Identification of the signals SpxR responds to is further complicated by its domain architecture, which is unusual for a prokaryotic TF. It contains several predicted ligand-binding domains (DRTGG, tandem CBS and HotDog; **S1B Fig**). We therefore anticipate several metabolites to interact with SpxR, adding another level of regulatory complexity to *cps* transcriptional control. Given the conservation of SpxR and its multipronged domain architecture in firmicutes [43,44], one exciting prospect is that our studies here could translate to other Gram-positives. Identifying the ligand(s) of both SpxR and CpsR, and the regulatory consequences of ligand binding will be the focus of future studies.

The distance of the *37-CE* from the core promoter elements and our EM structural data raises the question of how SpxR repression may be mediated mechanistically. One example in the literature that might help us understand this is the LrpC/AsnC family of TFs, which make a uniform wheel-like tetramer of dimers (*i*.*e*. are octameric). This configuration then interacts with both distal and proximal binding sites to regulate promoter activity [45,46]. Interestingly, when comparing the SpxR trimeric wheel structure with that of Lrp, despite the large discrepancy between quaternary units (SpxR is a trimer and Lrp is an octamer) they show strikingly similar diameters (**S6 Fig**). This phenomenon is due to the larger size of SpxR compared to Lrp (~48 kDa versus 16 kDa, respectively) and might suggest, in certain cases, functional conservation in a subset of distal TF regulatory control systems, irrespective of the TF’s monomeric molecular weight.

In our low resolution SpxR structure, it is not clear where the DNA is located. Along the three-fold axis the map is only around 88 Å in height, which would not cover the 37 base pairs (~ 120 Å) in the DNA fragment. Alternatively, as in the example of Lrp, the DNA could wrap around the SpxR trimer, however it is likely a 37-mer would only cover one of its three faces (**S6 Fig**). This observation would suggest that the precise mechanism by which SpxR (and CpsR) regulates *cps* expression could be complicated, and possibly involves the histone-like protein, HlpA, which is known to maintain pneumococcal chromosomal structure, [47] and both stabilizes and bends DNA to accommodate distal enhancer elements (*i*.*e*. the *37-CE*) in regulating transcription [48] (**Fig 7**). Indeed, HlpA was found to interact with the *cps* regulatory region and the *37-CE* in our initial DNA pull-down (**Fig 1**). The involvement of such a factor is supported by the ability of the *T>G 21-CE* variant to allow for derepression in the sepsis model (**Fig 4F**) despite intermediate changes in SpxR and CpsR’s affinity for the *21-CE* (**Fig 2C**). We envision that during infection, changes in metabolism driven by oxygen and sugar availability, in turn, drive changes in the concentration(s) of the as-of-yet unknown SpxR ligand(s) to facilitate pneumococcal lung-to-blood transition and sepsis, possibly including quaternary changes, to ultimately allow RNA polymerase and/or other TFs access to the *cps* core promoter/regulatory region (**Fig 7**). This phenomenon is exemplified in the LysR-type transcriptional regulator CrgA from *Neisseria meningitidis*, which forms ligand-induced hexadecameric rings that then interact with adjacent binding sites, thereby regulating promoter activity through DNA bending [49,50]. However, demonstrating if such a mechanism is responsible for SpxR regulation of the *cps* locus, and how HlpA and CpsR are integrated in this regulation remains to be determined.

The literature has demonstrated that the molecular makeup of the capsule itself, which varies among serotypes considerably, is a predictor of pneumococcal colonization and invasive behavior [14,15,51,52]. This phenomenon can, in part, be explained by the differential ability of liver Kupffer cells to capture different capsule serotypes during sepsis [15]. We predict that in addition to the molecular structure of the capsule, variation observed within the *37-CE* among serotypes (**Fig 6**) and, therefore, by inference, the regulatory control of the *cps* locus by SpxR and CpsR, could play an additional important role in serotype-specific capsule expression and tissue-specific pneumococcal virulence. Future experiments will determine the extent to which the *37-CE* influences colonization, transmission and pathogenicity between serotypes.

## Materials and methods

### Ethics statement

Mouse studies were performed under the project (permit no. PP0757060) and personal (permit no. 80/10279) licenses according to the United Kingdom Home Office guidelines under the Animals Scientific Procedures Act 1986, and the University of Leicester ethics committee approval. The protocol used was approved by both the U.K. Home Office and the University of Leicester ethics committee. Where specified, the procedures were done under anesthetic with isoflurane. Animals were kept in individually ventilated cages in a controlled environment and were regularly monitored after infection to reduce suffering.

### Growth conditions and transformation

For routine strain maintenance *S*. *pneumoniae* was grown statically at 37°C in a humidified incubator with 5% CO_2_, diluted 1:100 from -80°C stock into Todd-Hewitt broth (Oxoid, Oxford, UK) supplemented with 0.5% (w/v) yeast extract (Sigma-Aldrich, St Louis, MO) (THY). Culture density was measured at 600 nm using a Genesys 150 UV-vis spectrophotometer (Thermo Fisher Scientific, Waltham, MA). Antibiotics were not used in liquid cultures of *S*. *pneumoniae*. For experiments, C+Y (pH 6.8), a liquid casein-based medium supplemented with yeast extract (Sigma-Aldrich) was used as per Aprianto *et al*. [56].

Strains of *E*. *coli* were cultured for plasmid purification overnight with shaking at 230 rpm in a 37°C incubator in Luria-Bertani medium (LB, Difco; Beckton Dickinson, Franklin Lakes, NJ) supplemented with appropriate antibiotics: 50 μg/ml carbenicillin (Carb) or 50 μg/ml kanamycin (Kan).

For transformation of *S*. *pneumoniae*, cultures were grown as above until the an OD_600_ of 0.01–0.03 was reached. At this time, Competence-Stimulating Peptide 1 (CSP-1; Eurogentec, Seraing, Belgium) was added to a final concentration of 200 ng/ml. The culture was then incubated for 14 minutes before the addition of 100–200 ng DNA. After the addition of transforming DNA, cultures were returned to the CO_2_ incubator and incubated between 45 minutes and 2 hours before being plated onto tryptic soy broth (TSB, Criterion; Hardy Diagnostics, Santa Maria, CA) containing 5% (v/v) sheep blood (Lampire, Pipersville, PA), solidified with 1.5% agar (VWR, Radnor, PA) and supplemented with the appropriate antibiotics (TSBA). Plates were incubated at 37°C in the CO_2_ incubator until resistant colonies appeared. The following antibiotic concentrations were used for *S*. *pneumoniae*: 200 μg/ml streptomycin (Sm); 500 μg/ml Kan, and 3 μg/ml tetracycline (Tet).

### Preparation of starter cultures

Strains were streaked out from -80°C stocks onto TSBA containing the appropriate antibiotics and incubated at 37°C in a humidified CO_2_ incubator for between 24 and 48 hours. Then, THY broth was inoculated with a sweep of colonies and grown as above until the culture reached an OD_600_ of 0.4. Cultures were incubated on ice for 20 minutes before pelleting at 2000 x g for 10 minutes at 4°C. Pellets were resuspended in THY + 10% glycerol to an OD_600_ of 0.4 before storing at -80°C in 1 ml aliquots. One the day of use, starter cultures were thawed at 37°C for 90 seconds, cells were pelleted at 2000 x g for 5 minutes and then resuspended in the appropriate medium for inoculation.

### DNA affinity chromatography pulldown and mass spectrometry

To isolate and identify novel DNA-binding proteins of the *cps* promoter, a method described by Jutras *et al*. was used [21]. DNA probes were either PCR amplified from D39 genomic DNA using Phusion High-Fidelity polymerase (New England Biolabs (NEB), Ipswich, MA) followed by gel extraction of the resulting products with primer pair Pcps biotin F/Pcps biotin R, or synthesized as complementary forward and reverse primers (Sigma-Aldrich) and annealed at room temperature (37-CE biotin F/37-CE biotin R and 37-CEs biotin F/37-CEs biotin R; **S1 Table**). Forward primers were labelled with biotin and included a triethyleneglycol (TEG) spacer. For each DNA probe, 200 μl of M-280 Streptavidin Dynabeads (Invitrogen, Waltham, MA) were used. To prepare beads for binding of probe DNA, beads were washed three times and resuspended in 2X B/W buffer (10 mM Tris-Cl pH 7.5, 1 mM ethylenediaminetetraacetic acid (EDTA), 2 M NaCl) before rolling at room temperature with 40 μg of probe DNA. The bead-probe complexes were then washed 3 times in TE buffer (10 mM Tris-Cl pH 8.0, 1 mM EDTA) before washing twice with BS-THES buffer (22 mM Tris-Cl pH 7.5, 4.4 mM EDTA, 8.9% sucrose (m/v), 62 mM NaCl, 0.3% protease inhibitor cocktail II (Sigma-Aldrich; v/v), 0.04% phosphatase inhibitor cocktail II (Sigma-Aldrich; v/v), 10 mM HEPES, 5 mM CaCl_2_, 50 mM KCl, 12% glycerol). Beads were then washed with BS/THES buffer supplemented with 5 μg polydeoxyinosinic-deoxycytidylic acid Poly(dI-dC) (Sigma-Aldrich).

To prepare pneumococcal cell lysates, WT D39 was grown statically in THY medium at 37°C in a humidified incubator with 5% CO_2_, diluted 1:100 from starter cultures in a 1 L volume. Once the culture reached an OD_600_ of 0.35–0.45, it was placed on ice for 20 minutes before pelleting at 2,500 x g at 4°C. Cells were then washed twice with ice-cold MilliQ water and then the cell pellet was frozen at -80°C. After 3 freeze-thaw cycles, the cell pellet was resuspended in 2 ml ice-cold BS-THES buffer. Cells were then lysed using a Branson Sonifier S-450 cell disruptor (Branson Ultrasonics Corp., Danbury, CT) for a total of 2 minutes of sonication at 70% amplitude with 10 second pulses, separated by 1 minute rest periods. The temperature was maintained at or below 4°C by suspending the 2 ml tube in an ice bath. Cell debris was removed by centrifugation at 15,000 x g for 30 minutes at 4°C. 1.5 ml of the resulting supernatant was added to the prepared beads in the presence of 25 μg Poly(dI-dC) and rolled at room temperature for 30 minutes. The beads were then washed with BS/THES buffer containing 5 μg Poly dI-dC five times, followed by BS/THES buffer without Poly(dI-dC) twice. Bound proteins were eluted in increasing concentrations (100 mM, 200 mM, 300 mM, 500 mM, 750 mM and 1 M) of NaCl solution (100 μL) and frozen at -80°C before analysis by SDS-PAGE. Proteins were visualized using Coomassie and silver nitrate.

Protein identification by mass spectrometry was carried out by the Institute of Biochemistry and Biophysics at the Polish Academy of Sciences, University of Warsaw. Both individual protein bands and whole eluates were analyzed.

### Construction of plasmids

Plasmids used in this study are listed in **S1 Table**.

pPP3 is derived from pPP2, a *S*. *pneumoniae* suicide vector that allows for the integration of promoter-*lacZ* fusions at the *bgaA* locus (between and including SPD_0561 and SPD_0562—*bgaA*) [57]. To construct pPP3, the *CBRluc* gene codon-optimized for Gram-positive expression—a kind gift from Willem M. de Vos, University of Helsinki, Finland—preceded by a strong ribosome binding site (RBS) was amplified by PCR and ligated into pPP2 digested with *BamH*I and *Blp*I (NEB) using a restriction-less cloning method [58]. Two separate PCRs were run to generate *BamH*I and *Blp*I cohesive ends using primer pairs CBRluc F1/CBRluc R2 and CBRluc F2/CBRluc R1 (**S1 Table**). A map of pPP3 along with the multiple cloning site (MCS) sequence is shown in **S3 Fig**.

To generate pPP3 P*cps* luciferase reporter constructs, 289/252 bp fragments representing the upstream region of the *cps* operon (-282 to +16) without the native RBS of *cpsA* (SPD_0315) were amplified and ligated into pPP3 digested with *BamH*I and *Kpn*I (NEB). The *cps* regulatory region was amplified using either WT *S*. *pneumoniae* D39 or the relevant P_*cps*_ 37/21-CE variant genomic DNA as template. Two separate PCRs were run to generate *BamH*I and *Kpn*I cohesive ends using primer pairs Pcps F1/Pcps R1 and Pcps F2/Pcps R2 (**S1 Table**).

To generate pET15DG1-CpsA_ED_ we first predicted the ectodomain of CpsA (SPD_0315 amino acids 98–481) using JPred 4 [59]. The *cpsA*_*ED*_ DNA fragment was then amplified from *S*. *pneumoniae* D39 genomic DNA by PCR and ligated into pET15DG1 digested with *Nde*I and *BamH*I (NEB). *Nde*I and *BamH*I cohesive ends were generated by combining the products of reactions using primer pairs CpsA_ED_ F1/ CpsA_ED_ R2 and CpsA_ED_ F2/ CpsA_ED_ R1 (**S1 Table**).

To generate pE-SUMO-SpxR, pE-SUMO-SpxR_DBD_, and pE-SUMO-CpsR, *spxR* and *cpsR* sequences were amplified from *S*. *pneumoniae* D39 genomic DNA by PCR and ligated into pE-SUMO digested with *Bsa*I (NEB). The DNA-binding domain (DBD) of SpxR (amino acids 1–63) was predicted using JPred 4 [59]. Two separate PCRs were run to generate *Bsa*I cohesive ends for each insert: SpxR F1/SpxR R2 and SpxR F2/SpxR1; SpxR F1/SpxR_DBD_ R2 and SpxR F2/ SpxR_DBD_ R1; and CpsR F1/CpsR R2 and CpsR F2/CpsR R1 (**S1 Table**).

All plasmids were verified by Sanger sequencing.

### Strain construction

Bacterial strains used in this study are listed in (**S1 Table**).

Markerless mutations were introduced into the chromosome by a two-step transformation procedure using the Janus construct, *kan-rpsL*^*+*^ [60]. Using the Sm^R^ D39 *rpsL* strain, the Janus construct can first be inserted by homologous recombination into the locus of interest by selecting for Kan^R^ transformants. The resulting strain is Kan^R^ but Sm^S^ due to the *rpsL*^*+*^ allele in the Janus construct. In the second transformation step, the Janus construct is replaced with the desired sequence following selection with Sm. The resulting strain is Sm^R^ and Kan^S^. DNA fragments for transformation were generated by overlap-extension PCR.

To construct the P_*cps*_ *37/21-CE* variants, the *37-CE* in the *cps* promoter (upstream of *cpsA—*SPD_0315) was first replaced with the Janus construct; three DNA fragments were generated and fused by PCR using primer pairs 37-CE lift F/37-CE up R1, 37-CE-J F/37-CE-J R and 37-CE down F1/37-CE lift R using D39 *ritR*::*kan-rpsL*^*+*^ genomic DNA as template (**S1 Table**). The resulting product was transformed into D39 *rpsL* to generate D39 37-CE-*kan-rpsL*^*+*^. Next, two DNA fragments were generated and fused using primer pairs 37-CE lift F/37-CE up R2 and 37-CE down F2/37-CE lift R; 37-CE lift F/T>G up R and T>G down F/37-CE lift R; 37-CE lift F/TA>GT up R and TA>GT down F/37-CE lift R; 37-CE lift F/TAT>GAG up R and TAT>GAG down F/37-CE lift R; 37-CE lift F/SpxR only up R and SpxR only down F/37-CE lift R; 37-CE lift F/CpsR only up R and CpsR only down F/37-CE lift R; and 37-CE lift F/Neither up R and Neither down F/37-CE lift R (**S1 Table**). The resulting products were transformed into D39 37-CE-*kan-rpsL*^*+*^to create strains D39 Δ*37-CE*, D39 *T>G*, D39 *TA>GT*, D39 *TAT>GAG*, D39 *SpxR-only*, D39 *CpsR-only* and D39 *Neither*.

To construct D39 Δ*cps*, the *cps* locus was first replaced with the Janus construct; three DNA fragments were generated and fused by PCR using primer pairs cps lift F/cps up R1, cps-J F/cps-J R and cps down F1/cps lift R using D39 *ritR*::*kan-rpsL*^*+*^ genomic DNA as template (**S1 Table**). The resulting product was transformed into D39 *rpsL* to generate D39 *cps*::*kan-rpsL*^*+*^. Next, two DNA fragments were generated and fused using primer pairs cps lift F/cps up R2 and cps down F2/cps lift R (**S1 Table**). The resulting product was transformed into D39 *cps*::*kan-rpsL*^*+*^ to generate D39 Δ*cps*. This strain retains the first three codons of *cpsA* (SPD_0315) and the last three codons of *cpsO* (SPD_0331).

To construct D39 Δ*spxR*, the *spxR* locus (SPD_0969) was first replaced with the Janus construct; three DNA fragments were generated and fused by PCR using primer pairs spxR lift F/spxR-J up R1, spxR-J F/spxR-J R and spxR down F1/spxR lift R using D39 *ritR*::*kan-rpsL*^*+*^ genomic DNA as template (**S1 Table**). The resulting product was transformed into D39 *rpsL* to generate D39 *spxR*::*kan-rpsL*^*+*^. Next, two fragments were generated and fused using primer pairs spxR lift F/spxR up R2 and spxR down F2/spxR lift R (**S1 Table**). The resulting product was transformed into D39 *spxR*::*kan-rpsL*^*+*^ to generate D39 Δ*spxR*. This strain retains the first eleven and last three codons of *spxR*, thereby maintaining the stop codon of the overlapping gene encoded upstream.

To construct D39 Δ*cpsR*, the *cpsR* locus (SPD_0064) was first replaced with the Janus construct; three DNA fragments were generated and fused by PCR using primer pairs cpsR lift F/cpsR up R1, cpsR-J F/cpsR-J R and cpsR down F1/spxR lift R using D39 *ritR*::*kan-rpsL*^*+*^ genomic DNA as template (**S1 Table**). The resulting product was transformed into D39 *rpsL* to generate D39 *cpsR*::*kan-rpsL*^*+*^. Next, two DNA fragments were generated and fused using primer pairs cpsR lift F/cpsR up R2 and cpsR down F2/cpsR lift R (**S1 Table**). The resulting product was transformed into D39 *cpsR*::*kan-rpsL*^*+*^ to generate D39 Δ*cpsR*. This strain retains the first and last three codons of *cpsR*.

At least 6 clones of each mutant were isolated and tested for growth similarity before moving forward with a single clone for experiments. All mutations were verified by Sanger sequencing using the corresponding check primers (**S1 Table**).

To construct luciferase reporter strains, PP3 P_*cps*_ reporter plasmids were transformed into D39 *rpsL* and correct integration was confirmed by PCR using pPP3-tet-F/pPP3-tet-R and pPP3-bga-F/pPP3-bga-R (**S1 Table**). Luciferase activity of six individual clones of each reporter strain were tested to ensure consistency, and one clone was selected for all subsequent experimentation.

### Protein expression and purification

Protein expression plasmids were transformed into *E*. *coli* T7 Express lysY^*i/q*^ (**S1 Table**). Cultures were grown at 37°C with shaking at 230 rpm in LB medium supplemented with either 50 μg/ml carbenicillin (pET15DG1) or 50 μg/ml kanamycin (pE-SUMO/pFGET19_Ulp1). Expression of His_6_-tagged proteins was induced by the addition of 1 mM isopropyl-ß-D-1-thiogalactopyranoside (IPTG; GoldBio, St. Louis, MO) when cultures reached an OD_600_ of ~0.8. The temperature was then reduced to 18°C and the cultures were grown overnight with shaking at 150 rpm. Cells were harvested by centrifugation at 2000 x g for 20 minutes at 4°C. Pellets were stored at -80°C until use.

For purification of SpxR (full-length and DBD) and CpsR, pellets were resuspended in 25 ml/liter of culture of lysis/wash buffer (50 mM Tris-Cl pH 7.0, 500 mM NaCl, 40 mM imidazole, 1 mM ß-mercaptoethanol (BME)) supplemented with 1 mM phenylmethylsulfonyl fluoride (PMSF). Cells were then lysed by sonication using a Branson Sonifier S-450 cell disruptor (Branson Ultrasonics Corp., Danbury, CT) for a total of 3 minutes of sonication at 70% amplitude with 20 second pulses, separated by 5-minute rest periods in an ice bath. Lysates were clarified by centrifugation at 27,000 x g for 45 minutes at 4°C. The supernatant was filtered using 0.45 μm filters before being applied to 4 ml Ni-nitrilotriacetic acid (NTA)-agarose (Qiagen, Valencia, CA) by gravity flow to isolate His_6_-tagged proteins. Contaminating proteins were removed by washing the column with 100 ml lysis/wash buffer. His_6_-tagged proteins were eluted in 50 ml elution buffer (50 mM Tris-Cl pH 7.0, 500 mM NaCl, 250 mM imidazole, 1 mM BME) in 1 ml fractions. The purity of fractions was assessed by SDS-PAGE and Coomassie staining before pooling. 1 mM EDTA was added prior to dialysis against 2 L of dialysis buffer (50 mM Tris-Cl pH 7.0, 500 mM NaCl, 1 mM BME) overnight at 4°C to remove imidazole. Recombinant proteins were then brought to room temperature before cleavage of the His_6_-SUMO tag by addition of 0.1 mg/ml Ulp1 for 1 hour. Cleavage reactions were applied to Ni-NTA resin by gravity flow to remove His_6_-SUMO/His_6_-Ulp1; the cleaved protein is collected in the flowthrough. The cleaved protein was then concentrated to ~ 15 mg/ml using centrifugal filters (Amicon; Sigma-Aldrich) and clarified by centrifugation at 21,000 x g at room temperature for 10 minutes in preparation for SEC using a Cytiva Äkta Pure 25 chromatography system. 2 ml of the clarified protein was applied to a HiPrep 26/60 Sephacryl S-300 HR column (26 mm x 600 mm; Cytiva, Marlborough, MA) equilibrated in FPLC buffer (50 mM Tris-Cl pH 7.0, 500 mM NaCl, 1 mM TCEP) at a flow rate of 0.5 ml/minute at room temperature. Peak fractions were analyzed by SDS-PAGE and Coomassie staining before pooling and dialysis against storage buffer (50 mM Tris-Cl pH 7.0, 500 mM NaCl, 1 mM TCEP, 10% glycerol) overnight at room temperature. Proteins were flash-frozen in liquid nitrogen before storage at -80°C. The resulting preparations were >95% pure. Protein concentration was determined using Bradford reagent (Bio-Rad Laboratories, Hercules, CA), using the included bovine serum albumin (BSA) to generate a standard curve.

Purification of CpsA ectodomain (CpsA_ED_) was performed using the above protocol with some differences. Buffers were at pH 8.0 rather than pH 7.0 and included 150 mM NaCl instead of 500 mM. CpsA_ED_ has an N-terminal His_6_-tag which was removed using TEV protease (NEB) according to the manufacturer’s instructions.

### Estimation of molecular weights by size exclusion chromatography (SEC)

SEC experiments were carried out using a Cytiva Äkta Pure 25 chromatography system equipped with a Superose 6 Increase 10/300 GL (10 mm x 300 mm; Cytiva) equilibrated with 50 mM Tris-Cl pH 7.0, 500 mM NaCl, 1 mM TCEP (FPLC buffer). The column was calibrated using Gel Filtration Marker Kit (MWGF1000; Sigma-Aldrich—carbonic anhydrase 29 kDa, albumin 66 kDa, alcohol dehydrogenase 150 kDa, ß-amylase 200 kDa, apoferritin 443 kDa). Samples were applied (300 μL at 70 μM) to the column and separated at a flow rate of 0.4 ml/min.

### Electrophoretic mobility shift assays (EMSAs)

6-carboxyfluorescein (6-FAM)-labelled oligonucleotides, as well as a biotin-labelled scrambled oligo (non-fluorescent negative control oligo) were used in EMSA experiments (**S1 Table**). The *cps* regulatory region (-282 to +16) was amplified by PCR from D39 *rpsL* genomic DNA using primer pair Pcps 6-FAM F/Pcps biotin R. For all other EMSAs, complementary oligonucleotides were annealed with a 10% excess of the unlabeled oligonucleotide. Binding reactions were carried out in 10 μL volumes containing 20 mM HEPES pH 7.2, 50 mM NaCl, 5 mM MgCl_2_, 1 mM CaCl_2_, 0.1 mM EDTA, 12% glycerol, 10 mM DTT, 0.5 μM oligonucleotide, 400 ng Poly(dI-dC) and protein concentrations between 0 and 5 μM. Where necessary, competition oligonucleotides were added at 10X concentration (5 μM). Reaction mixtures were resolved using 8% non-denaturing 1X Tris-Glycine EDTA (TGE) gels. Gels were visualized using Cy2 filters (472 nm excitation, 513 nm emission) with an Azure 400 imaging system (Azure Biosystems. Inc, Dublin, CA).

### Fluorescence polarization (FP)

6-carboxyfluorescein (6-FAM)-labelled oligonucleotides were used in fluorescence polarization experiments (**S1 Table**). Binding reactions were carried out in 150 μL volumes containing 20 mM HEPES pH 7.2, 50 mM NaCl, 5 mM MgCl_2_, 1 mM CaCl_2_, 0.1 mM EDTA, 2% glycerol, 10 nM oligonucleotide and protein concentrations between 0 and 1.25 μM. For high-salt conditions the same buffer was used except the final concentration of NaCl was 500 mM. After a 10 minute equilibration, 45 μL of each binding reaction was measured in triplicate in a black 384-well plate (781900; Greiner Bio-One, Stonehouse, UK) using a BioTek Synergy HI multimode plate reader (BioTek, Winooski, VT) equipped with the green FP filter set (8040561; excitation 485/20 nm, emission 528/20 nm, dichroic mirror 510 nm). Polarization values were calculated using the Biotek Gen5 software. Equilibrium dissociation constants were calculated using a one-site binding model (total binding) in GraphPad Prism version 9.3.1 for mac (GraphPad Software, San Diego, CA).

### Luciferase assays

To measure luciferase activity, *S*. *pneumoniae* luciferase reporter strains were grown in C+Y medium (pH 6.8) diluted 1:10 from starter culture (see above). Once cultures reached the mid-exponential phase (OD_600_ 0.3–0.5), they were placed on ice for 10 minutes to halt growth, then the OD_600_ was measured. Luciferase reactions were started by addition of 10 μL of D-Luciferin potassium salt (GoldBio) dissolved in MilliQ water at 10 mM concentration to 140 μL culture (0.667 mM final concentration of D-Luciferin) in a white 96-well plate (Greiner Bio-One, Stonehouse, UK). Luminescence was measured after a 15 minute incubation in the luminometer (Veritas microplate luminometer, Turner BioSystems) with a 1 second integration time. Three biological replicates were performed and three technical replicates were measured for each biological replicate. The luminescence was normalized to the OD_600_ of the culture and is expressed as Relative Light Units (RLU = Luminescence/OD_600_).

### Western blot analysis

To measure CpsA protein levels, *S*. *pneumoniae* P_*cps*_ *37/21-CE* variants were grown in C+Y medium (pH 6.8) diluted 1:10 from starter culture (see above). Once cultures reached the mid-exponential phase (OD_600_ 0.3–0.5), they were placed on ice for 10 minutes to halt growth, then the OD_600_ was measured. 2 ml of culture was pelleted at 10,000 x g for 2 minutes at 4°C and frozen at -20°C until use. Pellets were resuspended in 2X Laemmli buffer according to the equation: volume of 2X Laemmli = OD_600_ of culture x volume of culture x 100. Cells were then boiled at 95°C for 5 minutes. Protein extracts were separated by SDS-PAGE and then transferred to PVDF membranes (EMD Millipore). Rabbit-raised antiserum against CpsA_ED_ was prepared by Covance Inc. (Princeton, NJ) by immunization of 6–8 week old specific pathogen-free rabbits three times at three-weekly intervals with purified recombinant CpsA ectodomain (CpsA_ED_; see above). Specificity of the antiserum was verified by western blot of protein extracts from D39 *rpsL* and D39 Δ*cps* strains. The pre-immune bleed was also tested. Rabbit-raised antisera against PiaA was originally prepared by CoalAb inc. (UK) and was a generous gift from Dr. Jeremy Brown, University College London, UK. Anti-PiaA IgG was purified using a Protein A column as described in ref (61). Anti-CpsA anti-serum was used at a dilution of 1:50,000 and Anti-PiaA at 1:10,000. Proteins of interest were detected using HRP-conjugated goat anti-rabbit IgG (1:5000; Jackson ImmunoResearch, UK) and visualized with Pierce ECL Western Blotting substrate (Thermo Scientific) and Azure 400 Imaging System (Azure Biosystems). Western blots were quantified using ImageJ [62]

### Measurement of capsule thickness by FITC-dextran exclusion assay

The thickness of the capsule in the WT and 37/*21-CE* variants was determined by measuring the exclusion of 2,000-kDa fluorescein isothiocyanate (FITC)-dextran (FD20000S-100MG; Sigma-Aldrich) using a variation of the method of Hamaguchi *et al*. [63]. *S*. *pneumoniae* P_*cps*_ *37/21-CE* variants were grown in C+Y medium (pH 6.8) diluted 1:10 from starter culture (see above) until the culture reached mid-exponential phase (OD_600_ 0.3–0.5). 1 ml of each culture was pelleted and washed with phosphate-buffered saline (pH 7.4; PBS). Finally, the pellets were resuspended in 1 ml of PBS. 90 μl of the resuspension was mixed well with 10 μl of 10 mg/ml FITC dextran. 8 μl of the cell suspension was transferred onto glass slides previously coated with poly-L-lysine (Sigma-Aldrich) then covered with a coverslip. The slides were imaged at 63X magnification using a Leica DMI4000B inverted spinning disk confocal microscope (Leica Microsystems, Wetzlar, Germany) equipped with a Q-imaging Retiga EXi 12-bit cooled camera (Qimaging, Surrey, BC, Canada). Phase-contrast and FITC images were acquired and processed using MetaMorph software (Molecular Devices, San Jose, CA). Capsule thickness was quantified using the ImageJ measure tool [62]. First, the width of the cell in the FITC image was measured (black edges), then the width of the cell was measured in the phase image. The phase value (cell width) was subtracted from the FITC value (capsule width) and divided by two to give the capsule thickness. Images from five biological replicates were analyzed.

### Size Exclusion Chromatography and Multiangle Light Scattering (SEC-MALS)

Solution SEC-MALS experiments were conducted in the Keck Biophysics Facility at Northwestern University (Evanston, IL) using an Agilent Technologies 1200 LC system (Agilent Technologies, Santa Clara, CA) equipped with Wyatt Dawn Heleos II 18-angle MALS and Wyatt QELS light scattering detectors, Optilab T-rEX refractive index detector and ASTRA 5.3.4.20 software (Wyatt Technology Europe GmbH, Dernbach, Germany). In accordance to the manufacturer’s guidelines, the Dawn Heleos II detector was calibrated with toluene and the Optilab T-rEX detector was calibrated with sodium chloride standards. The size exclusion column—Superdex 200 increase 10/300 GL (Cytiva)—was equilibrated overnight in buffer at 0.4 ml/min flow rate at 25°C. A void volume of 7.8 ml was determined using blue dextran (Sigma-Aldrich).

Samples were prepared in 50 mM Tris-Cl pH 7.0, 500 mM NaCl, 1 mM TCEP at a concentration of 70 μM and cleared of aggregates by filtration through 0.22 um Millipore centrifugal filters. Injection volumes were 200 μls. To calibrate the system and to monitor its performance, a 200 μl injection of bovine serum albumin (BSA) standard (1.0 mg/ml) dissolved in the same buffer was performed and analyzed. The BSA monomer’s molecular weight was determined to be 66.6 KDa, in excellent agreement with the theoretical value of 66.4 KDa. In each run, the protein concentration was determined using an average refractive index increment (dn/dc) of 0.179 ml·g −1 at the laser wavelength (658 nm), Data analysis was performed using ASTRA 5.3.4.20 software as described in ref. (64).

### Negative stain electron microscopy

To prepare samples for negative stain EM analysis of high salt SpxR with DNA the complex was made by mixing 1 μl of protein with 50-fold excess DNA and incubated at room temperature for 5 minutes. 1.5 μl of this mixture was diluted with 50 μl of buffer consisting of 50 mM Tris pH 7.0, 500 mM NaCl, 1mM TCEP. Carbon-coated copper EM grids (400 mesh Cu-UL, Electron Microscopy Sciences, Hatfield, PA) were glow-discharged for 10 seconds at 15 W in a Pelco easiGLOW plasma cleaner (Ted Pella, Inc., Altadena, CA). Glow-discharged grids were placed carbon side down onto a 30 μl drop of the diluted SpxR/DNA sample for 2 minutes at room temperature and subsequently transferred twice to 30 μl drops of water for 1 minute per drop, with blotting occurring after the second transfer. The grids were then transferred twice to 30 μl drops of 1.5% uranyl acetate solution for 30 seconds per drop, followed by blotting after the second transfer and finally allowing the grid to air dry. To prepare low salt SpxR with DNA samples for negative stain EM analysis the DNA stock (500 μM) was diluted 10 fold in water; 2 μl of the DNA solution was added to 2 μl of 1 mg/ml SpxR in 50 mM Tris pH 7.0, 500 mM NaCl, 1mM TCEP, and incubated at room temperature for 30 minutes. This was followed by dilution with 36 μl of 50 mM HEPES, pH 7.5, 5 mM MgCl_2_, 1 mM CaCl_2_. After dilution of SpxR in this buffer, the final NaCl concentration was 50 mM. Low salt reactions were then incubated for 45 minutes at room temperature. Grids were prepared as above, but glow discharge was increased to 60 seconds at 20 W. In both cases, EM data were collected on a JEOL 1400 transmission electron microscope (TEM; JEOL, Peabody, MA) operating at 120 keV with an UltraScan4000 CCD camera using the program SerialEM [65] at a nominal magnification of 50,000x with a pixel size of 2.2 Å at the specimen level, and using a defocus range of -1.5 μm to -2.5 μm. For the high salt data, 399 micrographs were collected while 1,112 micrographs were used for the low salt specimen. Micrographs were processed using RELION 4 [66] and cryoSPARC [67]. In both cases, particles were picked automatically and successive rounds of 2D classification were used to remove noise and bad particles yielding 48,739 and 18,974 particles for high and low salt alone data sets, respectively. In each case, the selected particles were used to generate *ab initio* models. Visual inspection of the class averages and the resulting models indicated C2 and C3 symmetry for the high and low salt model, respectively, and this symmetry was imposed on the final refinement steps. The nominal resolution of these two models are 20.9 Å and 21.5 Å for the high and low salt structures, respectively.

### Colonization, pneumonia and sepsis models of infection

The effect of *37*/*21-CE* variants on pneumococcal *in vivo* survival and virulence was tested using mouse models of colonization, pneumonia, and sepsis. For these models, 9–11 weeks old outbred, female mice bred in the Division of Biomedical Services at the University of Leicester were used.

For the colonization model, each mouse was infected intranasally with 5x10^5 CFU in 20 μl PBS (pH 7.0) intranasally under light anesthesia using 2.5% (v/v) isoflurane (Isocare) over oxygen (1.4–1.6 liters/minute). While applying the dose, mice were kept horizontally to prevent dissemination of the inoculum into the lungs. At predetermined time points, mice were killed and their nasopharyngeal content was recovered using 0.5 ml PBS. The colony counts were determined by plating serial dilutions of the nasopharyngeal wash.

For the pneumonia model, mice were infected intranasally with 5x10^6 CFU/mouse in 50 μl PBS under light anesthesia using 2.5% (v/v) isoflurane (Isocare) over oxygen (1.4–1.6 liters/minute). Mice were kept vertically while applying the dose dropwise into each nostril. At 24 hours post-infection, a small sample of blood was taken from the tail vein to determine the pneumococcal load in blood by plating. Mice were followed for the signs of disease (hunched, piloerect, or lethargic) over 168 hours. When they reached the severely lethargic stage they were considered to have reached the endpoint of the assay and were killed humanely. The time to reach a lethargic state was considered as “survival time.” Mice that were alive 168 hours after infection were considered to have survived the infection. In addition, to determine the lung counts of the strains, mice were killed after 24 hours post intranasal infection. Lungs were taken and homogenized in 10 mls of PBS and the pneumococcal counts were determined by plating the serial dilutions of lung homogenates.

The impact of mutations in the sepsis model was also tested. 2x10^6 CFU/mouse in 100 μl PBS was administered via tail vein. The disease signs were monitored over 120 hours and the survival time was recorded as above. The pneumococcal counts were determined by plating blood samples obtained from the tail vein at 24 and 48 hours post-infection

### *In vivo* imaging

The influence of *37*/*21-CE* variants on P_*cps*_ activity during infection was determined using the P_*cps*_::*CBRluc* reporter strains. 9–11 weeks old outbred, female mice bred in the Division of Biomedical Services at the University of Leicester were infected with 5x10^6 CFU in 50 μl PBS under light anesthesia using 2.5% (v/v) isoflurane over oxygen (1.4–1.6 liters/min). Approximately 4 hours post-infection, mice were anesthetized and administered subcutaneously with luciferin (150 mg/kg). The animals were imaged using the IVIS Spectrum *in vivo* imaging system (Perkin Elmer, Waltham, MA) over 20 minutes with one-minute intervals. Immediately after imaging, mice were killed to dissect their lungs and the pneumococcal counts were determined in the lung homogenates to normalize the signal levels against the colony-forming units.

### Bioinformatics

Sequences of the *cps* region from 87 serotypes were obtained from the EBML server [33]. Alignments were generated using Clustal Omega [53]. Phylogenetic trees were generated by uploading Clustal output files into iTOL [55]. SpxR binding motifs were initially predicted by uploading curated *21-CE* and *37-CE* sequences into Meta-Meme motif searching program [54]. To identify the ACTAKWMTAGA motif (p value of 2.5 x 10^−5^) a maximum of 3 and minimum of 2 motifs, and maximum of 15 nucleotides per motif found, were used as the settings.

### Statistics

Statistical analysis was performed using GraphPad Prism version 9.3.1 for mac (Graphpad Software, San Diego, CA). Statistical tests used for each experiment are indicated in the figure legends. Measurements were made in technical replicates and at least three biological replicates were performed for each experiment.

## Supporting information

S1 Fig Additional *Pcps* interacting protein data. (**A**) MS statistics of the 5 identified transcription factors from the 750 mM NaCl eluate are shown. (**B**) Domain architectures of identified P_*cps*_ interacting factors. BHD, Bacterial Histone-like Domain; HTH, Helix-Turn-Helix; CBS, cystathionine beta-synthase; LBD, Ligand Binding Domain. (**C**) SDS-PAGE of recombinant full-length SpxR and CpsR (left) and SpxR_DBD_ (right). (**D**) Representative SEC chromatogram demonstrating that SpxR is a tetramer, CpsR is a dimer, and SpxR_DBD_ is a monomer in solution. *Inset*: molecular weight (MW) standards used for mass calculations.
(PDF)

S2 Fig Additional SpxR_DBD_ and CpsR EMSAs. (**A**) SpxR_DBD_ EMSAs and (**B**) CpsR EMSAs. For reference, the *cps* promoter from **Fig 2A** is shown (**C**). The precise *37-CE/21-CE* double stranded DNA oligos used in experiments are shown above the gel shifts. To define the minimal sequences required for SpxR and CpsR interaction within the *37/21-CE* a series of *37-CE* truncations were tested for SpxR and CpsR interaction using EMSA. It was first hypothesized that the interactions occurred at either the 10 bp inverted repeats highlighted in light blue, or within the 17 bp spacer region highlighted in pink. Neither SpxR nor CpsR interacted with these regions alone, indicating that they must occur around the junctions of these sequences. Therefore, we tested interactions with oligos consisting of the 10 bp inverted repeat regions (light blue) that had been extended by either 5 or 10 nucleotides (37 CE 5’ + 5/10 and 37 CE 3’ + 5/10). For SpxR_DBD_, interaction was strongest with the 37 CE 5’ + 10 oligo, followed by the 37 CE 3’ + 10 oligo. Closer examination of these two sequences identified two similar inverted repeat sequences, one that is imperfect on the 5’ half of the *37-CE* and another that is perfect on the 3’ half. These are underlined in red for the 37 CE 5’ IR/3’ IR oligos. To achieve robust interaction, the 37 CE 5’ IR and 3’ IR oligos had to be extended (37 CE 5/3’ IR +3/4 each end). We have defined the *spxR1* and *spxR2* sites as these two inverted repeats within the *37-CE* (see C). Unlike SpxR, CpsR was found to only interact with the 5’ end of the *37-CE* (see shifts with 37 CE 5’ + 10 and 37-CE 3’ + 10). We hypothesize that CpsR interacts with the direct repeat underlined in black in the 21 CE oligo shift (also depicted in C). Making the *spxR1* site a perfect inverted repeat (21 CE perfect oligo) dramatically reduces the affinity of interaction between CpsR and the 21 CE. Gels are representative of 3 independent experiments. (**D**) Fluorescence polarization with CpsR. We were unable to obtain sigmoidal binding curves with CpsR, likely due to its small size in solution (dimer, 65.72 kDa) relative to the 21-CE oligo. Experiments were carried out as per Fig 2 using protein concentrations between 0 and 5 μM.
(JPG)

S3 Fig pPP3 *CBRluc* luciferase reporter vector. (**A**) Schematic diagram of pPP3 luciferase reporter plasmid. Multiple Cloning Site (MCS) DNA sequence is shown above with unique restriction sites, save *EcoR*I which has two cut sites. (**B**) Click beetle luciferese (*CBRluc*) expression and enzymatic activity driven by the D39 capsule promoter (P_*cps*_) does not affect pneumococcal growth. Growth of P*cps*::*CBRluc* reporter induced (+) and uninduced (-) with luciferin (*inse*t: luciferase activity during log phase growth). Plasmid map was generated using SnapGene software (Insightful Science, San Diego, CA).
(JPG)

S4 Fig Growth curves of *21-CE* mutants. Representative growth curves of wild-type D39 and *37/21-CE* isogenic D39 mutant strains cultured in C+Y medium (pH 6.8) in 5% CO_2_ under static conditions.
(JPG)

S5 Fig CpsR EMSAs with potential ligands. (**A**) (Above) The *21-CE* sequence with the potential CpsR binding site underlined. (*Left*) Addition of various potential CpsR ligands or glucose/glucose-6-phosphate (*right*) to EMSAs at 1000x concentration over that of the CpsR protein (5 mM sugar to 5 uM protein). (**B**) (*Above*) Annotated schematic diagram of the P_*cps*_ showing the *37-CE* and core promoter. (*Below*) Increasing concentrations of CpsR in the presence of the full-length P_*cps*_. Glucose was added in the last lane at 1000X the concentration of CpsR (5 mM glucose to 5 uM protein).
(JPG)

S6 Fig The low salt SpxR conformation could accommodate multiple binding sites. The figure shows a comparison between the negative stain EM map of the low salt SpxR “wheel” conformation, a model of a 37-mer (*37-CE*) B-DNA molecule, and the structure of the octamer of the Lrp/AsnC transcriptional regulator (4 dimers; PDB ID 1i1q (Leonard et al., 2001)). Lrp/AsnC is a smaller transcriptional regulator with several DNA binding sites forming a multimeric structure. The diagram shows that parallel to the 3-fold axis the SpxR trimer is shorter than a straight B-DNA 37-mer (bottom panels), but also that the 37-mer is too short to interact simultaneously with more than one of the faces orthogonal to the 3-fold axis (top panels).
(JPG)

S1 Table Plasmids, strains and oligonucleotides used in this study. (DOCX)
