# Supplementary figures and images for: Pneumococcal capsule expression is controlled through a conserved, distal cis-regulatory element during infection
Source: PLoS Pathog. 2023 Jan 31;19(1):e1011035. doi: 10.1371/journal.ppat.1011035 (PMC9888711; doi:10.1371/journal.ppat.1011035)

Figure S1

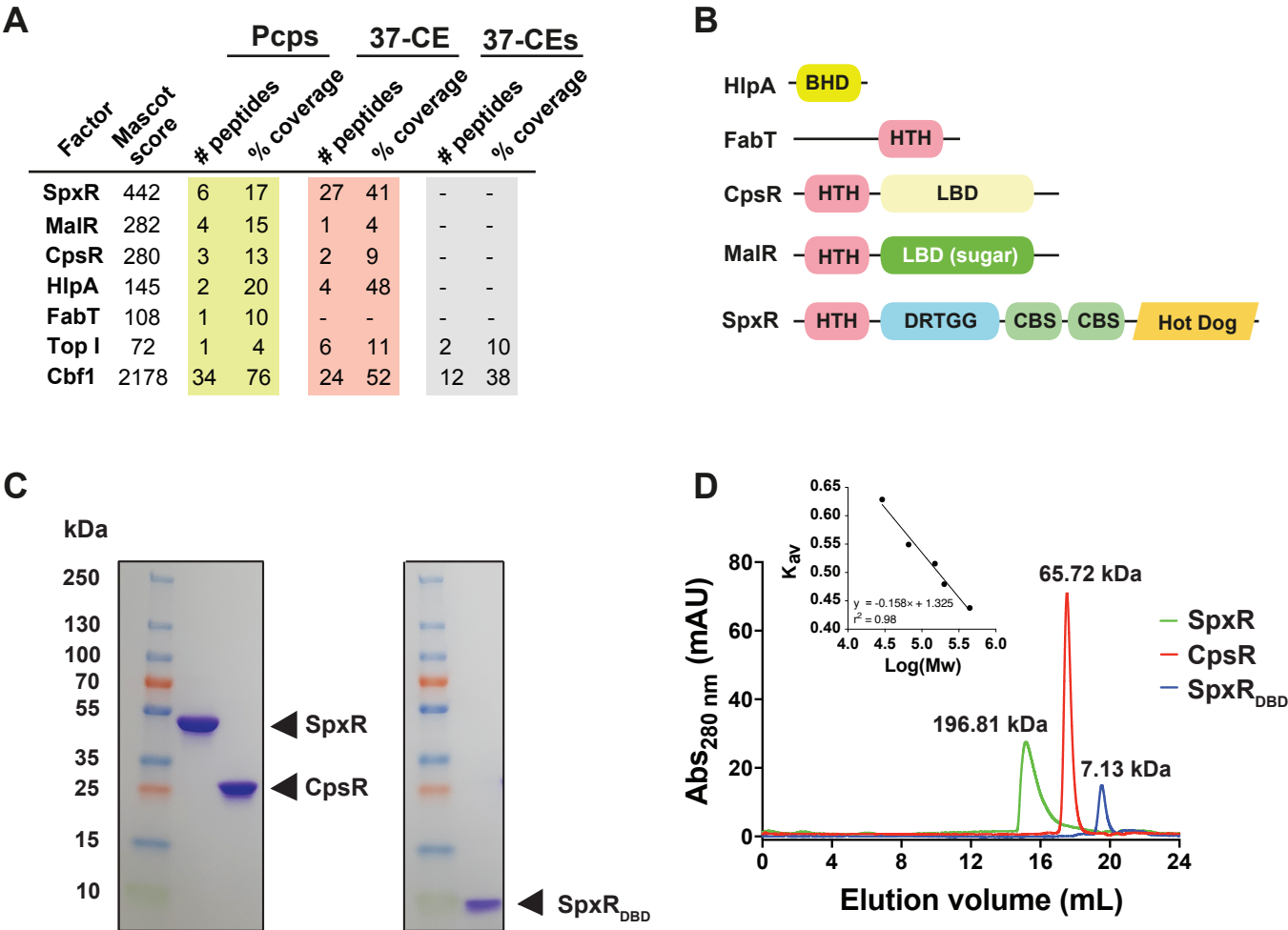

Supplement: S1 Fig — (A) MS statistics of the 5 identified transcription factors from the 750 mM NaCl eluate are shown. (B) Domain architectures of identified Pcps interacting factors. BHD, Bacterial Histone-like Domain; HTH, Helix-Turn-Helix; CBS, cystathionine beta-synthase; LBD, Ligand Binding Domain. (C) SDS-PAGE of recombinant full-length SpxR and CpsR (left) and SpxRDBD (right). (D) Representative SEC chromatogram demonstrating that SpxR is a tetramer, CpsR is a dimer, and SpxRDBD is a monomer in solution. Inset: molecular weight (MW) standards used for mass calculations. (PDF) [file ppat.1011035.s001.pdf]

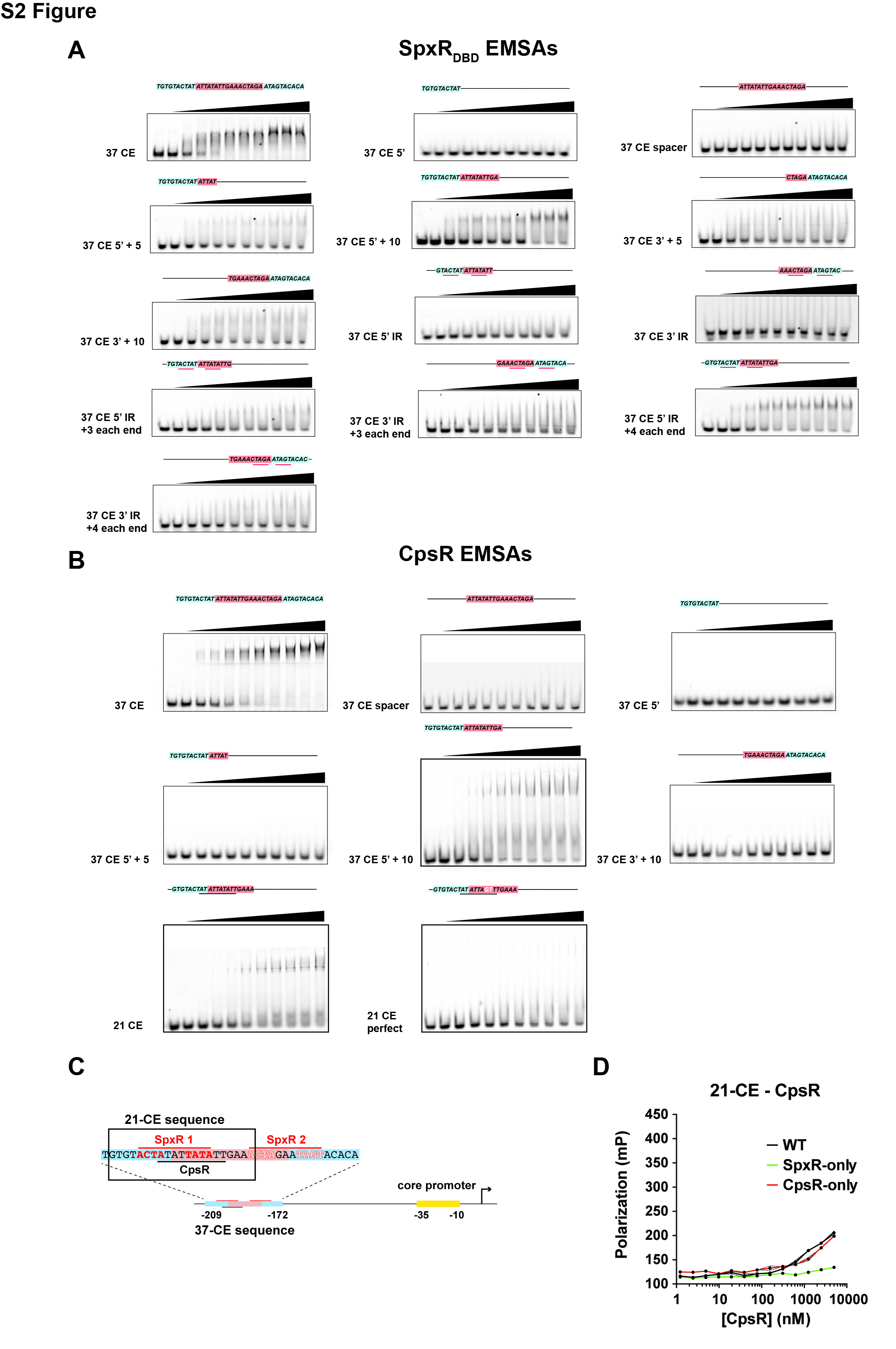

Supplement: S2 Fig — (A) SpxRDBD EMSAs and (B) CpsR EMSAs. For reference, the cps promoter from Fig 2A is shown (C). The precise 37-CE/21-CE double stranded DNA oligos used in experiments are shown above the gel shifts. To define the minimal sequences required for SpxR and CpsR interaction within the 37/21-CE a series of 37-CE truncations were tested for SpxR and CpsR interaction using EMSA. It was first hypothesized that the interactions occurred at either the 10 bp inverted repeats highlighted in light blue, or within the 17 bp spacer region highlighted in pink. Neither SpxR nor CpsR interacted with these regions alone, indicating that they must occur around the junctions of these sequences. Therefore, we tested interactions with oligos consisting of the 10 bp inverted repeat regions (light blue) that had been extended by either 5 or 10 nucleotides (37 CE 5’ + 5/10 and 37 CE 3’ + 5/10). For SpxRDBD, interaction was strongest with the 37 CE 5’ + 10 oligo, followed by the 37 CE 3’ + 10 oligo. Closer examination of these two sequences identified two similar inverted repeat sequences, one that is imperfect on the 5’ half of the 37-CE and another that is perfect on the 3’ half. These are underlined in red for the 37 CE 5’ IR/3’ IR oligos. To achieve robust interaction, the 37 CE 5’ IR and 3’ IR oligos had to be extended (37 CE 5/3’ IR +3/4 each end). We have defined the spxR1 and spxR2 sites as these two inverted repeats within the 37-CE (see C). Unlike SpxR, CpsR was found to only interact with the 5’ end of the 37-CE (see shifts with 37 CE 5’ + 10 and 37-CE 3’ + 10). We hypothesize that CpsR interacts with the direct repeat underlined in black in the 21 CE oligo shift (also depicted in C). Making the spxR1 site a perfect inverted repeat (21 CE perfect oligo) dramatically reduces the affinity of interaction between CpsR and the 21 CE. Gels are representative of 3 independent experiments. (D) Fluorescence polarization with CpsR. We were unable to obtain sigmoidal binding curves with CpsR, [file ppat.1011035.s002.jpg]

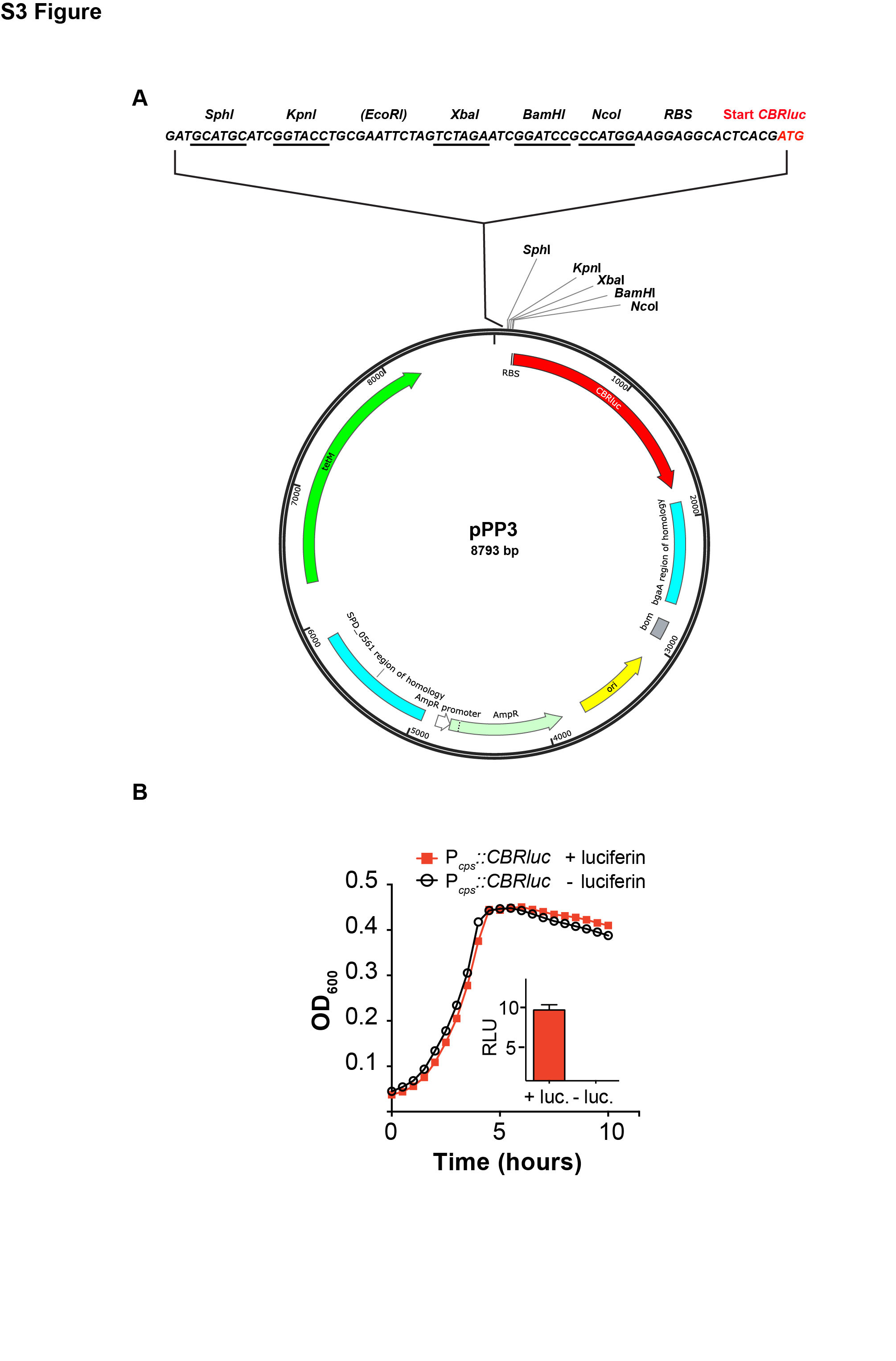

Supplement: S3 Fig — (A) Schematic diagram of pPP3 luciferase reporter plasmid. Multiple Cloning Site (MCS) DNA sequence is shown above with unique restriction sites, save EcoRI which has two cut sites. (B) Click beetle luciferese (CBRluc) expression and enzymatic activity driven by the D39 capsule promoter (Pcps) does not affect pneumococcal growth. Growth of Pcps::CBRluc reporter induced (+) and uninduced (-) with luciferin (inset: luciferase activity during log phase growth). Plasmid map was generated using SnapGene software (Insightful Science, San Diego, CA). (JPG) [file ppat.1011035.s003.jpg]

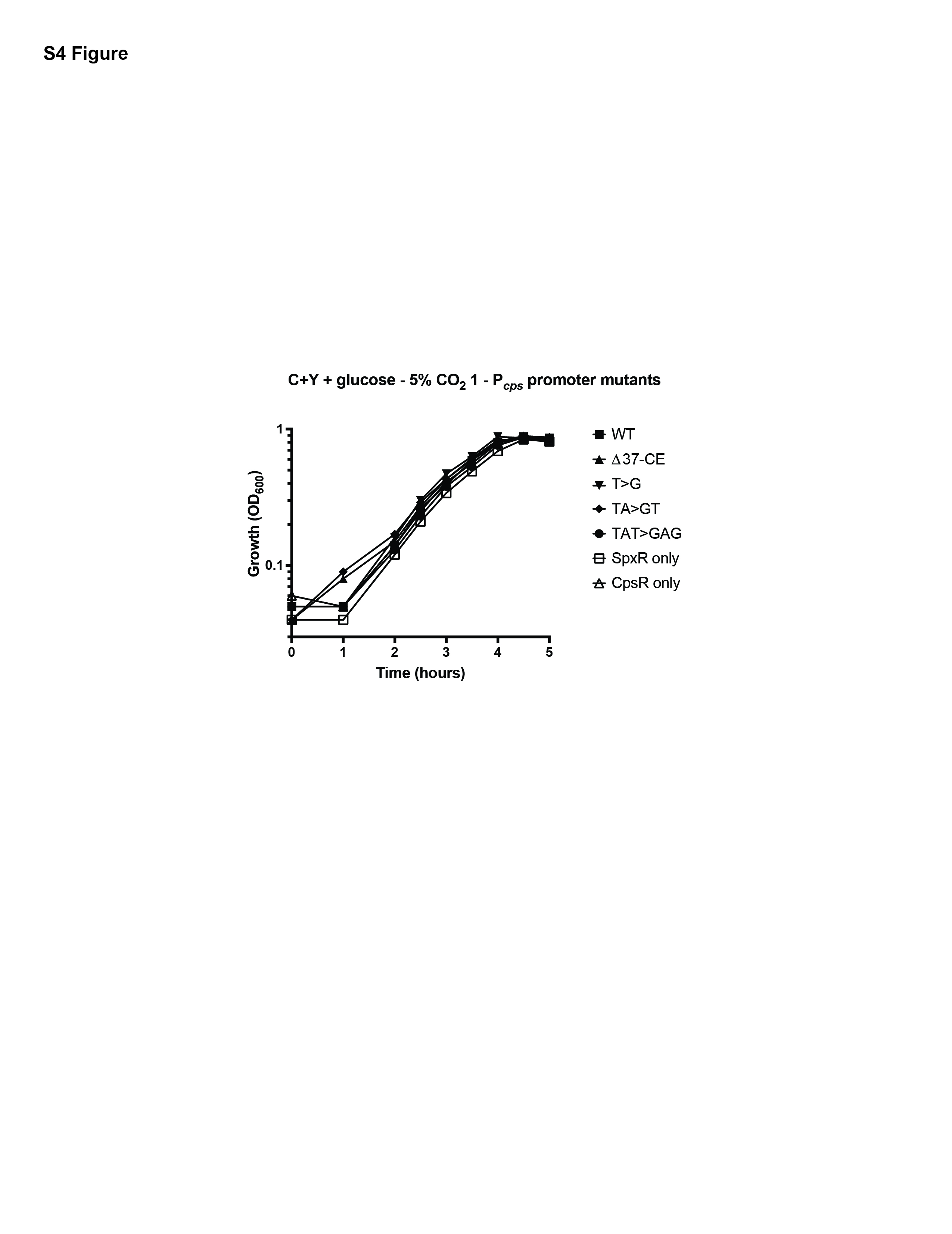

Supplement: S4 Fig — Representative growth curves of wild-type D39 and 37/21-CE isogenic D39 mutant strains cultured in C+Y medium (pH 6.8) in 5% CO2 under static conditions. (JPG) [file ppat.1011035.s004.jpg]

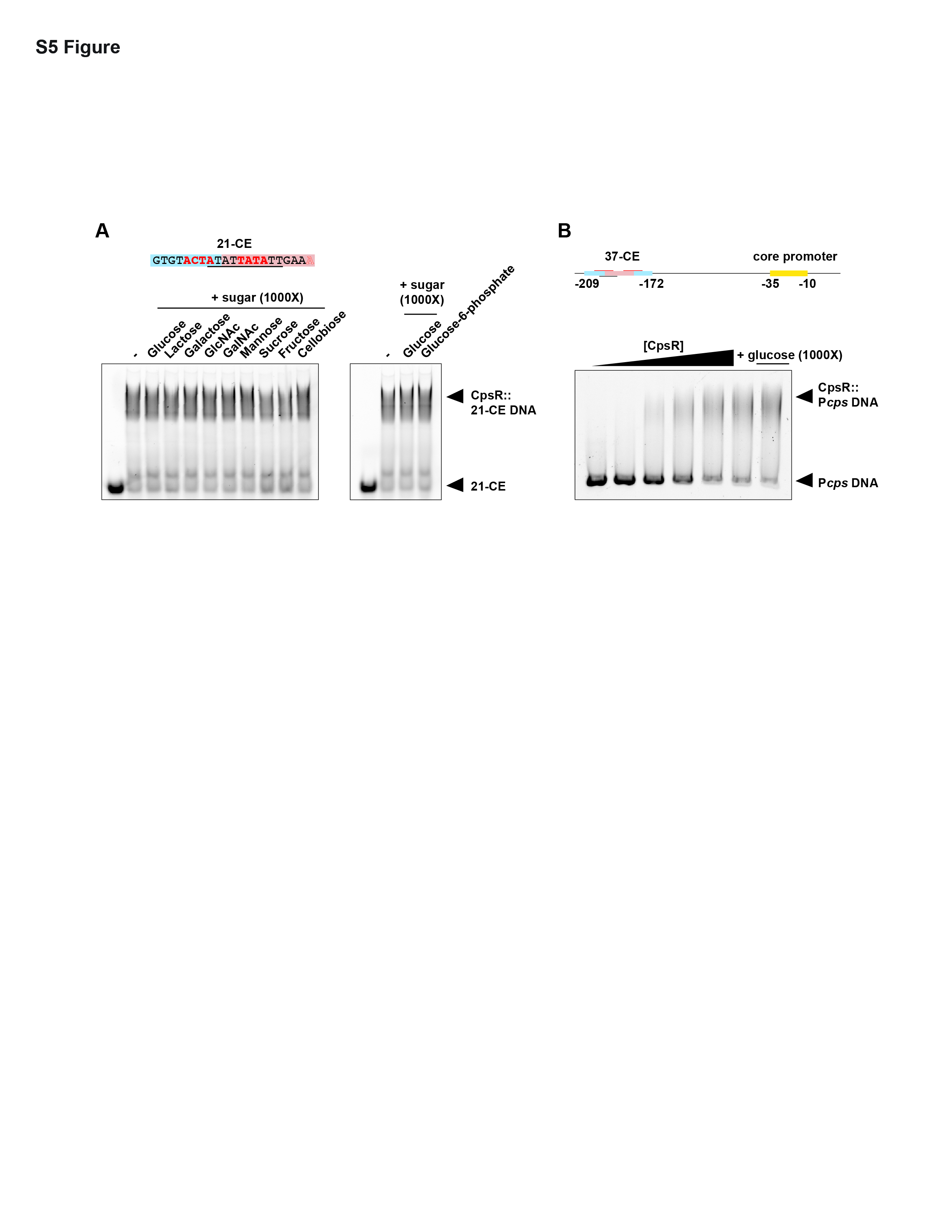

Supplement: S5 Fig — (A) (Above) The 21-CE sequence with the potential CpsR binding site underlined. (Left) Addition of various potential CpsR ligands or glucose/glucose-6-phosphate (right) to EMSAs at 1000x concentration over that of the CpsR protein (5 mM sugar to 5 uM protein). (B) (Above) Annotated schematic diagram of the Pcps showing the 37-CE and core promoter. (Below) Increasing concentrations of CpsR in the presence of the full-length Pcps. Glucose was added in the last lane at 1000X the concentration of CpsR (5 mM glucose to 5 uM protein). (JPG) [file ppat.1011035.s005.jpg]

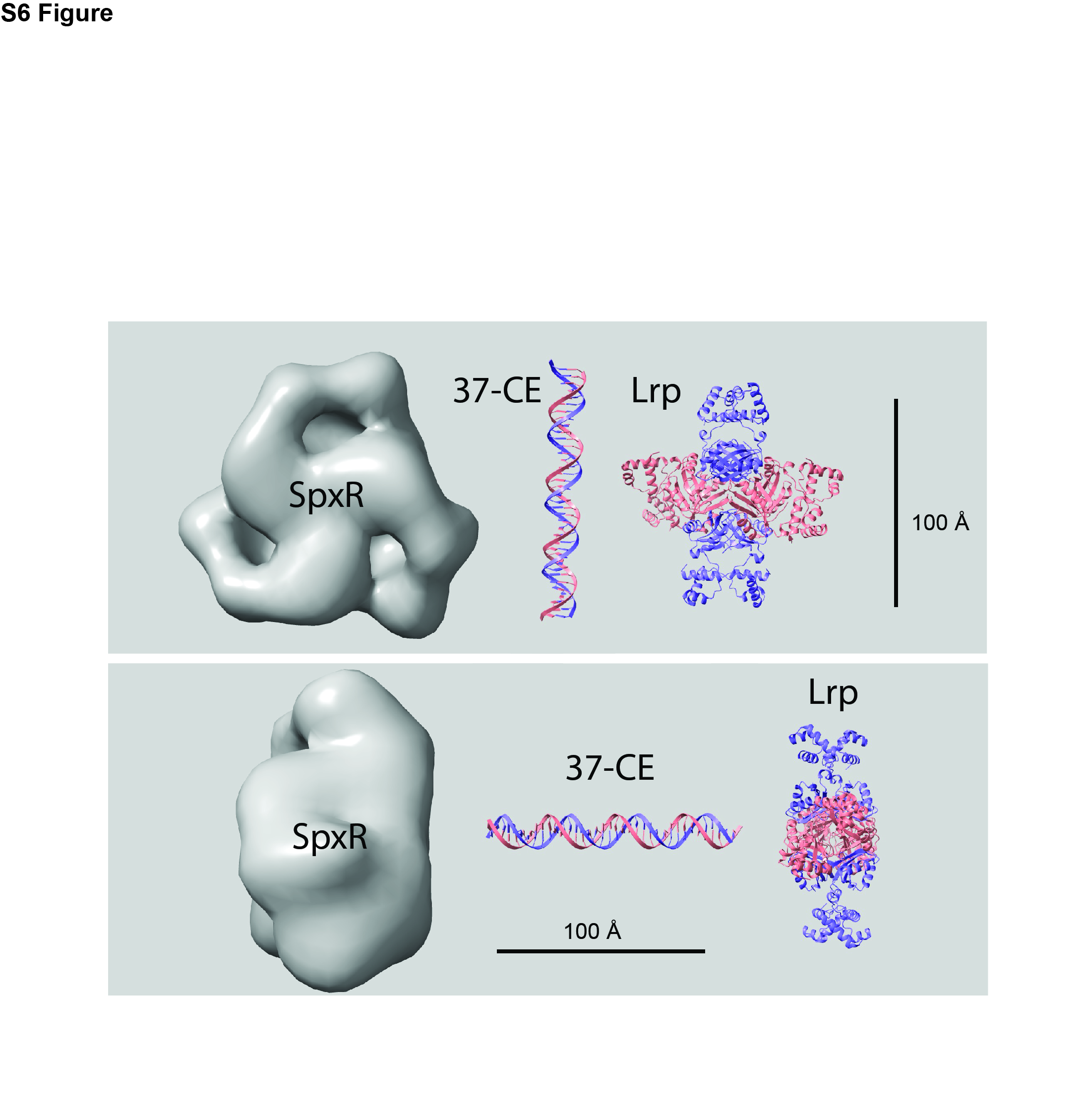

Supplement: S6 Fig — The figure shows a comparison between the negative stain EM map of the low salt SpxR “wheel” conformation, a model of a 37-mer (37-CE) B-DNA molecule, and the structure of the octamer of the Lrp/AsnC transcriptional regulator (4 dimers; PDB ID 1i1q (Leonard et al., 2001)). Lrp/AsnC is a smaller transcriptional regulator with several DNA binding sites forming a multimeric structure. The diagram shows that parallel to the 3-fold axis the SpxR trimer is shorter than a straight B-DNA 37-mer (bottom panels), but also that the 37-mer is too short to interact simultaneously with more than one of the faces orthogonal to the 3-fold axis (top panels). (JPG) [file ppat.1011035.s006.jpg]
