# Supplementary material for: Pneumococcal capsule expression is controlled through a conserved, distal cis-regulatory element during infection
Source: PLoS Pathog. 2023 Jan 31;19(1):e1011035. doi: 10.1371/journal.ppat.1011035 (PMC9888711; doi:10.1371/journal.ppat.1011035)
Supplement: S1 Table — (DOCX) [file ppat.1011035.s007.docx]

| **S1 Table. Plasmids, strains and oligonucleotides used in this study** | | |
| --- | --- | --- |
| **Plasmid** | **Description** | **Source or reference** |
| pE-SUMO | N-terminal His_6_-SUMO fusion protein expression vector. Kan^R^. | Life Sensors Inc. |
| pE-SUMO-SpxR | pE-SUMO containing SpxR (SPD_0969). Kan^R^. | This work |
| pE-SUMO-SpxR_DBD_ | pE-SUMO containing the DNA-binding domain of SpxR (DBD - amino acids 1 - 63). Kan^R^. | This work |
| pE-SUMO-CpsR | pE-SUMO containing CpsR (SPD_0064). Kan^R^. | This work |
| pFGET19_Ulp1 | Vector for expression of His_6_-tagged Ulp1 (SUMO protease). IPTG-inducible. Kan^R^. | (1) |
| pET15DG1 | Vector for expression of His_6-_tagged proteins. Contains TEV cleavage site. Amp^R^. | (2) |
| pET15DG1-CpsA_ED_ | pET15DG1 containing the predicted ectodomain (ED - amino acids 98 - 481) of CpsA (SPD_0315). Amp^R^. | This work |
| pPP2 | *S. pneumoniae bgaA* integrative plasmid with promoter-less *lacZ.* Amp^R^ (*E. coli*) and Tet^R^ (*S. pneumoniae*). | (3) |
| pPP3 | pPP2 with *lacZ* replaced with *CBRluc* from *Pyrophorus plagiopthalamus*. Amp^R^ (*E. coli*) and Tet^R^ (*S. pneumoniae*). | This work |
| pPP3-P*_cps-_*_WT_ | pPP3 containing WT *cps* promoter. Amp^R^ (*E. coli*) and Tet^R^ (*S. pneumoniae*). | This work |
| pPP3-P*_cps_*_-Δ37-CE_ | pPP3 containing "Δ37-CE" *cps* promoter variant. Amp^R^ (*E. coli*) and Tet^R^ (*S. pneumoniae*). | This work |
| pPP3-P*_cps_*_-SpxR only_ | pPP3 containing "SpxR-only" *cps* promoter variant. Amp^R^ (*E. coli*) and Tet^R^ (*S. pneumoniae*). | This work |
| pPP3-P*_cps_*_-CpsR only_ | pPP3 containing CpsR-only *cps* promoter variant. Amp^R^ (*E. coli*) and Tet^R^ (*S. pneumoniae*). | This work |
| pPP3-P*_cps_*_-Neither_ | pPP3 containing "Neither" *cps* promoter variant. Amp^R^ (*E. coli*) and Tet^R^ (*S. pneumoniae*). | This work |

**Abbreviations**

Kan^R^ = Kanamycin resistant, Amp^R^ = Ampicillin resistant, Tet^R^ = Tetracycline resistant

| **Strain** | **Genotype or description** | **Source or reference** |
| --- | --- | --- |
| ***E. coli*** | | |
| DH5α | Cloning and plasmid maintenance strain. | Lab stock |
| T7 express *lysY^i/q^* | Protein expression strain. | New England Biolabs, Ipswich, MA |
| T7 express *lysY^i/q^* SUMO-SpxR | T7 express *lysY^i/q^* His_6_-SUMO-SpxR expression strain. Kan^R^. | This work |
| T7 express *lysY^i/q^* SUMO-SpxR_DBD_ | T7 express *lysY^i/q^* His_6_-SUMO-SpxR_DBD_ expression strain. Kan^R^. | This work |
| T7 express *lysY^i/q^* SUMO-CpsR | T7 express *lysY^i/q^* His_6_-SUMO-CpsR expression strain. Kan^R^. | This work |
| T7 express *lysY^i/q^* Ulp1 | T7 express *lysY^i/q^* His_6_-Ulp1 expression strain. Amp^R^. | This work |
| ***S. pneumoniae*** | | |
| D39 *rpsL* | Strain NTCC 7466, encapsulated serotype 2 strain carrying the L56T mutation in *rpsL*. Sm^R^. | (4) |
| D39 *ritR::kan-rpsL^+^* | D39 *rpsL* with *kan-rpsL^+^* cassette in the *ritR* locus. Kan^R^. Sm^S^. | (4) |
| D39 *spxR::kan-rpsL^+^* | D39 *rpsL* with *kan-rpsL^+^* cassette in the *spxR* locus. Kan^R^. Sm^S^. | This work |
| D39 Δ*spxR* | Unmarked deletion of *spxR* in the D39 *rpsL* background. Mutant retains the first eleven and last three codons of the *spxR* gene. Sm^R^. | This work |
| D39 *cpsR::kan-rpsL^+^* | D39 *rpsL* with *kan-rpsL^+^* cassette in the *cpsR* locus. Kan^R^. Sm^S^. | This work |
| D39 Δ*cpsR* | Unmarked deletion of *cpsR* in the D39 *rpsL* background. Mutant retains the first and last three codons of the *cpsR* gene. Sm^R^. | This work |
| D39 *cps::kan-rpsL^+^* | D39 *rpsL* with *kan-rpsL^+^* cassette in the *cps* locus. Kan^R^. Sm^S^. | This work |
| D39 Δ*cps* | Unmarked deletion of the *cps* locus in the D39 *rpsL* background. Mutant retains the first and last three codons of the *spxR* gene. Sm^R^. | This work |
| D39 P*_cps_::kan-rpsL^+^* | D39 *rpsL* with *kan-rpsL^+^* cassette in the *cps* promoter region. Kan^R^. Sm^S^. | This work |
| D39 Δ37-CE | Unmarked deletion of the 37-CE in the D39 *rpsL* background. Sm^R^. | This work |
| D39 T>G | Unmarked P*_cps_* promoter variant with T>G 37-CE sequence in the D39 *rpsL* background. Sm^R^. | This work |
| D39 TA>GT | Unmarked P*_cps_* promoter variant with TA>GT 37-CE sequence in the D39 *rpsL* background. Sm^R^. | This work |
| D39 TAT>GAG | Unmarked P*_cps_* promoter variant with the TAT>GAG 37-CE sequence in the D39 *rpsL* background. Sm^R^. | This work |
| D39 SpxR only | Unmarked P*_cps_* promoter variant with the SpxR only variant 37-CE sequence in the D39 *rpsL* background. Sm^R^. | This work |
| D39 CpsR only | Unmarked P*_cps_* promoter variant with the CpsR only variant 37-CE sequence in the D39 *rpsL* background. Sm^R^. | This work |
| D39 Neither | Unmarked P*_cps_* promoter variant with the Neither variant 37-CE sequence in the D39 *rpsL* background. Sm^R^. | This work |
| D39 P*_cps_::CBRluc* WT | D39 *rpsL bgaA::tetM-P_cps-WT_::CBRluc.* Sm^R^. Tet^R^. | This work |
| D39 P*_cps_::CBRluc* Δ37-CE | D39 *rpsL bgaA::tetM-P_cps-_*_Δ37-CE_*::CBRluc.* Sm^R^. Tet^R^. | This work |
| D39 P*_cps_::CBRluc* SpxR only | D39 *rpsL bgaA::tetM-P_cps-_*_SpxR only_*::CBRluc.* Sm^R^. Tet^R^. | This work |
| D39 P*_cps_::CBRluc* CpsR only | D39 *rpsL bgaA::tetM-P_cps-Cps_*_R only_*::CBRluc.* Sm^R^. Tet^R^. | This work |
| D39 P*_cps_::CBRluc* Neither | D39 *rpsL bgaA::tetM-P_cps-Neither_::CBRluc.* Sm^R^. Tet^R^. | This work |

**Abbreviations**

Kan^R^ = Kanamycin resistant, Amp^R^ = Ampicillin resistant, Sm^R^ = Streptomycin resistant, Sm^S^ = Streptomycin sensitive, Tet^R^ = Tetracycline resistant.

|  | **Oligonucleotide name** | **Nucleotide sequence (5'-3')** | **Modification** |
| --- | --- | --- | --- |
| **DNA-affinity chromatography pulldown** | | | |
|  | Pcps biotin F | ACCTGTTCGCTCACAATAAGAGAG | Biotin-TEG |
|  | Pcps biotin R | CATCTTACGATTATATCACTTTTTTACGG |  |
|  | 37-CE biotin F | GGAAAACAAAAATGTGTACTATATTATATTGAAACTAGAATAGTACACAAAAAACCTGTGTACTATATTATATTGAAACTAGAATAGTACACAAAAAACC | Biotin-TEG |
|  | 37-CE biotin R | GGTTTTTTGTGTACTATTCTAGTTTCAATATAATATAGTACACAGGTTTTTTGTGTACTATTCTAGTTTCAATATAATATAGTACACATTTTTGTTTTCC |  |
|  | 37-CE_S_ biotin F | GGAAAACAAAAAATTAGATACCTGGTGTGGATTTAGTATTCCATATGATAAAAACCATTAGATACCTGGTGTGGATTTAGTATTCCATATGATAAAAACC | Biotin-TEG |
|  | 37-CE_S_ biotin R | GGTTTTTATCATATGGAATACTAAATCCACACCAGGTATCTAATGGTTTTTATCATATGGAATACTAAATCCACACCAGGTATCTAATTTTTTGTTTTCC |  |
| **Protein expression constructs** | | | |
|  | SpxR F1 | AGTTATGAGTAAGCATCAGGAAATTCTAAGC |  |
|  | SpxR F2 | ATGAGTAAGCATCAGGAAATTCTAAGC |  |
|  | SpxR R1 | CTAGCCCTAATTAATTTTAACAGTCACATTTGCTTTTGAAAC |  |
|  | SpxR R2 | CCCTAATTAATTTTAACAGTCACATTTGCTTTTGAAAC |  |
|  | SpxR_DBD_ R1 | CTAGCCTTACTGGGATTTAACACGAATTGTTCC |  |
|  | SpxR_DBD_ R2 | CCTTACTGGGATTTAACACGAATTGTTCC |  |
|  | CpsR F1 | AGTTATGGAGAAGGAAAATAGAGGTAAAAATATGG |  |
|  | CpsR F2 | ATGGAGAAGGAAAATAGAGGTAAAAATATGG |  |
|  | CpsR R1 | CTAGCCTTAGTGATCACGGTCACATGAGATG |  |
|  | CpsR R2 | CCTTAGTGATCACGGTCACATGAGATG |  |
|  | CpsA_ED_ F1 | TATGATGGGACTGACCAATCGTTTAAATGCGAC |  |
|  | CpsA_ED_ F2 | TGATGGGACTGACCAATCGTTTAAATGCGAC |  |
|  | CpsA_ED_ R1 | GATCCTCATCTACCCTCCATCACATCC |  |
|  | CpsA_ED_ R2 | CTCATCTACCCTCCATCACATCC |  |
| **Electrophoretic Mobility Shift Assays (EMSAs) & Fluorescence polarization** | | | |
|  | 37-CE FAM F | TGTGTACTATATTATATTGAAACTAGAATAGTACACA | 6-FAM |
|  | 37-CE F | TGTGTACTATATTATATTGAAACTAGAATAGTACACA |  |
|  | 37-CE R | TGTGTACTATTCTAGTTTCAATATAATATAGTACACA |  |
|  | 37-CE_s_ F | ATTAGATACCTGGTGTGGATTTAGTATTCCATATGAT |  |
|  | 37-CE_s_ R | ATCATATGGAATACTAAATCCACACCAGGTATCTAAT |  |
|  | Pcps 6-FAM F | ACCTGTTCGCTCACAATAAGAGAG | 6-FAM |
|  | 37-CE 5' 6-FAM F | TGTGTACTAT | 6-FAM |
|  | 37-CE 5' 6-FAM R | ATAGTACACA |  |
|  | 37-CE 6-FAM spacer F | ATTATATTGAAACTAGA | 6-FAM |
|  | 37-CE 6-FAM spacer R | TCTAGTTTCAATATAAT |  |
|  | 37-CE 6-FAM 5'+5 F | TGTGTACTATATTAT | 6-FAM |
|  | 37-CE 6-FAM 5'+5 R | ATAATATAGTACACA |  |
|  | 37-CE 6-FAM 3'+5 F | CTAGAATAGTACACA | 6-FAM |
|  | 37-CE 6-FAM 3'+5 R | TGTGTACTATTCTAG |  |
|  | 37-CE 6-FAM 5'+10 F | TGTGTACTATATTATATTGA | 6-FAM |
|  | 37-CE 6-FAM 5'+10 R | TCAATATAATATAGTACACA |  |
|  | 37-CE 6-FAM 3'+10 F | TGAAACTAGAATAGTACACA | 6-FAM |
|  | 37-CE 6-FAM 3'+10 R | TGTGTACTATTCTAGTTTCA |  |
|  | 37-CE 6-FAM 5' IR F | GTACTATATTATATT | 6-FAM |
|  | 37-CE 6-FAM 5' IR R | AATATAATATAGTAC |  |
|  | 37-CE 6-FAM 3' IR F | AAACTAGAATAGTAC | 6-FAM |
|  | 37-CE 6-FAM 3' IR R | GTACTATTCTAGTTT |  |
|  | 37-CE 6-FAM 5' IR+3 F | TGTACTATATTATATTG | 6-FAM |
|  | 37-CE 6-FAM 5' IR+3 R | CAATATAATATAGTACA |  |
|  | 37-CE 6-FAM 3' IR+3 F | GAAACTAGAATAGTACAC | 6-FAM |
|  | 37-CE 6-FAM 3' IR+3 R | GTGTACTATTCTAGTTTC |  |
|  | 37-CE 6-FAM 5' IR+4 F | GTGTACTATATTATATTGA | 6-FAM |
|  | 37-CE 6-FAM 5' IR+4 R | TCAATATAATATAGTACAC |  |
|  | 37-CE 6-FAM 3' IR+4 F | TGAAACTAGAATAGTACAC | 6-FAM |
|  | 37-CE 6-FAM 3' IR+4 R | GTGTACTATTCTAGTTTCA |  |
|  | 21-CE 6-FAM F | GTGTACTATATTATATTGAAA | 6-FAM |
|  | 21-CE 6-FAM R | TTTCAATATAATATAGTACAC |  |
|  | 21-CE T>G 6-FAM F | GTGTACTATATTAGATTGAAA | 6-FAM |
|  | 21-CE T>G 6-FAM R | TTTCAATCTAATATAGTACAC |  |
|  | 21-CE TA>GT 6-FAM F | GTGTACTATATTAGTTTGAAA | 6-FAM |
|  | 21-CE TA>GT 6-FAM R | TTTCAAACTAATATAGTACAC |  |
|  | 21-CE TAT>GAG 6-FAM F | GTGTACTAGAGTATATTGAAA | 6-FAM |
|  | 21-CE TAT>GAG 6-FAM R | TTTCAATATACTCTAGTACAC |  |
|  | 21-CE SpxR only 6-FAM F | GTGTACTAGAGTAGTTTGAAA | 6-FAM |
|  | 21-CE SpxR only 6-FAM F | TTTCAAACTACTCTAGTACAC |  |
|  | 21-CE CpsR only 6-FAM F | GTGTGAGATATTATATTGAAA | 6-FAM |
|  | 21-CE CpsR only 6-FAM R | TTTCAATATAATATCTCACAC |  |
|  | 21-CE Neither 6-FAM F | GTGTGAGAGAGTAGTTTGAAA | 6-FAM |
|  | 21-CE Neither 6-FAM R | TTTCAAACTACTCTCTCACAC |  |
| **pPP3** | | | |
|  | CBRluc F1 | GATCCGCCATGGAAGGAGGCACTCACGATGGTCAAGCGGGAAAAAAACGTTATTTAC |  |
|  | CBRluc F2 | CGCCATGGAAGGAGGCACTCACGATGGTCAAGCGGGAAAAAAACGTTATTTAC |  |
|  | CBRluc R1 | TCAGCCTAACCACCAGCTTTAACTAATAATTGC |  |
|  | CBRluc R2 | GCCTAACCACCAGCTTTAACTAATAATTGC |  |
|  | Pcps F1 | GTACCACCTGTTCGCTCACAATAAGAGAG |  |
|  | Pcps F2 | CACCTGTTCGCTCACAATAAGAGAG |  |
|  | Pcps R1 | GATCCCTATACATTGAACATCTTACGATTATATCAC |  |
|  | Pcps R2 | CCTATACATTGAACATCTTACGATTATATCAC |  |
| **Mutant constructs** | | | |
|  | spxR lift F | CGACCGACTTAAGAGCTGGG |  |
|  | spxR up R1 | GATTATATCACATTATCCATTAAAAATCAAACGGCAAATAGCTTAGAATTTCCTGATGCTTACTCAT |  |
|  | spxR-J F | ATGAGTAAGCATCAGGAAATTCTAAGCTATTTGCCGTTTGATTTTTAATGGATAATGTGATATAATC |  |
|  | spxR-J R | GTTATCATCTTTTCTCCTAGTTTCTAATTAATAGAGACCTGGGCCCCTTTC |  |
|  | spxR down F1 | GAAAGGGGCCCAGGTCTCTATTAATTAGAAACTAGGAGAAAAGATGATAAC |  |
|  | spxR lift R | CACCGTCAATGGTCTTATGCGC |  |
|  | spxR up R2 | GTTATCATCTTTTCTCCTAGTTTCTAATTAATCAAATAGCTTAGAATTTCCTGATGCTTACTCAT |  |
|  | spxR down F2 | ATGAGTAAGCATCAGGAAATTCTAAGCTATTTGATTAATTAGAAACTAGGAGAAAAGATGATAAC |  |
|  | cpsR lift F | CAACCTGCATCATGAGAATATTGCC |  |
|  | cpsR up R1 | GATTATATCACATTATCCATTAAAAATCAAACGGCTTCTCCATAGAAAATTTTACCATAAAAGCG |  |
|  | cpsR-J F | CCGTTTGATTTTTAATGGATAATGTGATATAATCCGCTTTTATGGTAAAATTTTCTATGGAGAAG |  |
|  | cpsR-J R | GCTCAGGCTTTCTTCTTAGTGATCAGAGACCTGGGCCCCTTTC |  |
|  | cpsR down F1 | GAAAGGGGCCCAGGTCTCTGATCACTAAGAAGAAAGCCTGAGC |  |
|  | cpsR lift R | CTGGTTTATCCTTGTCAGTCTTGTC |  |
|  | cpsR up R2 | GCTCAGGCTTTCTTCTTAGTGATCCTTCTCCATAGAAAATTTTACCATAAAAGCG |  |
|  | cpsR down F2 | CGCTTTTATGGTAAAATTTTCTATGGAGAAGGATCACTAAGAAGAAAGCCTGAGC |  |
|  | cps lift F | CATCGTCTCTTCGTAAGTCATTGG |  |
|  | cps up R1 | GATTATATCACATTATCCATTAAAAATCAAACGGGATTAACACCTATACATTGAACATCTTACG |  |
|  | cps-J F | CGTAAGATGTTCAATGTATAGGTGTTAATCCCGTTTGATTTTTAATGGATAATGTGATATAATC |  |
|  | cps-J R | CTCTTTTATTAGACTAGAAAATCATTCTACTACAGAGACCTGGGCCCCTTTC |  |
|  | cps down F1 | GAAAGGGGCCCAGGTCTCTGTAGTAGAATGATTTTCTAGTCTAATAAAAGAG |  |
|  | cps lift R | CTCTTCTTGACGTTTCTGTAGTCG |  |
|  | cps up R2 | CTCTTTTATTAGACTAGAAAATCATTCTACTACGATTAACACCTATACATTGAACATCTTACG |  |
|  | cps down F2 | CGTAAGATGTTCAATGTATAGGTGTTAATCGTAGTAGAATGATTTTCTAGTCTAATAAAAGAG |  |
|  | 37-CE lift F | GGATTGTACTCAGGTGAGTAGG |  |
|  | 37-CE up R1 | GATTATATCACATTATCCATTAAAAATCAAACGGATGTGGTATAATCTTTTTATGGCATATTCAATAG |  |
|  | 37-CE -J F | CTATTGAATATGCCATAAAAAGATTATACCACATCCGTTTGATTTTTAATGGATAATGTGATATAATC |  |
|  | 37-CE -J R | CAAATCGTTTTCTAACAATATTTTAGAAGCAGAAGAGACCTGGGCCCCTTTC |  |
|  | 37-CE down F1 | GAAAGGGGCCCAGGTCTCTTCTGCTTCTAAAATATTGTTAGAAAACGATTTG |  |
|  | 37-CE lift R | GACACTATTTAGGACAATGGCC |  |
|  | 37-CE up R2 | CAAATCGTTTTCTAACAATATTTTAGAAGCAGAATGTGGTATAATCTTTTTATGGCATATTCAATAG |  |
|  | 37-CE down F2 | CTATTGAATATGCCATAAAAAGATTATACCACATTCTGCTTCTAAAATATTGTTAGAAAACGATTTG |  |
|  | T>G up R | TGTGTACTATTCTAGTTTCAATCTAATATAGTACACAATGTGGTATAATCTTTTTATGGCATATTCAATAG |  |
|  | T>G down F | TGTGTACTATATTAGATTGAAACTAGAATAGTACACATCTGCTTCTAAAATATTGTTAGAAAACGATTTG |  |
|  | TA>GT up R | TGTGTACTATTCTAGTTTCAAACTAATATAGTACACAATGTGGTATAATCTTTTTATGGCATATTCAATAG |  |
|  | TA>GT down F | TGTGTACTATATTAGTTTGAAACTAGAATAGTACACATCTGCTTCTAAAATATTGTTAGAAAACGATTTG |  |
|  | TAT>GAG up R | TGTGTACTATTCTAGTTTCAATATACTCTAGTACACAATGTGGTATAATCTTTTTATGGCATATTCAATAG |  |
|  | TAT> GAG down F | TGTGTACTAGAGTATATTGAAACTAGAATAGTACACATCTGCTTCTAAAATATTGTTAGAAAACGATTTG |  |
|  | SpxR only up R | TGTGTACTATTCTAGTTTCAAACTACTCTAGTACACAATGTGGTATAATCTTTTTATGGCATATTCAATAG |  |
|  | SpxR only down F | TGTGTACTAGAGTAGTTTGAAACTAGAATAGTACACATCTGCTTCTAAAATATTGTTAGAAAACGATTTG |  |
|  | CpsR only up R | TGTGTACTATTCTAGTTTCAATATAATATCTCACACAATGTGGTATAATCTTTTTATGGCATATTCAATAG |  |
|  | CpsR only down F | TGTGTGAGATATTATATTGAAACTAGAATAGTACACATCTGCTTCTAAAATATTGTTAGAAAACGATTTG |  |
|  | Neither up R | TGTGTACTATTCTAGTTTCAAACTACTCTCTCACACAATGTGGTATAATCTTTTTATGGCATATTCAATAG |  |
|  | Neither down F | TGTGTGAGAGAGTAGTTTGAAACTAGAATAGTACACATCTGCTTCTAAAATATTGTTAGAAAACGATTTG |  |
| **Strain/plasmid verification and sequencing** | | | |
|  | spxR check F | TGCAACAACCGCTTACCCCT |  |
|  | spxR check R | ACGCATGATATCAGCCCCTAACACT |  |
|  | cpsR check F | GCATCTGCGAAAAGTTGTAAGGTGC |  |
|  | cpsR check R | TGATTTCCTTGAGCTGTTCTGGTGA |  |
|  | cps check F | GTAGGGAAGAAGAGGTAAAAGTTTATGC |  |
|  | cps check R | GTTCAGCTAGATTATTGGAAAGGACC |  |
|  | IR check 1 F | GTTGGATGACAGCCAAATTCAGAAG |  |
|  | IR check 1 R | GGACTAACGCAGTTACCACTAG |  |
|  | IR check 2 F | CTACATTTAACTATCTATTAGACTATTACGC |  |
|  | IR check 2 R | GAAAGACTGATTCTTAGACGTCTTAG |  |
|  | pPP3-tet-F | CCTAATCGGAAAGGTTTTCAATCCC |  |
|  | pPP3-tet-R | CCGATAACGATACCAAGGATGAAAC |  |
|  | pPP3-bga-F | GTCTTGGTTGGAACCAAACCAG |  |
|  | pPP3-bga-R | GTTTCAATCTACTATACAATAAGAGAACG |  |
|  | pPP3 seq F | GCATGCATCGGTACCTGCG |  |
|  | T7 F | TAATACGACTCACTATAGGG |  |
|  | T7 R | GCTAGTTATTGCTCAGCGG |  |

**Abbreviations**

TEG = Triethylene glycol, 6-FAM = 6-Carboxyfluorescein

**References**

1. Guerrero F, Ciragan A, Iwaï H. Tandem SUMO fusion vectors for improving soluble protein expression and purification. Protein Expr Purif. 2015;116:42-9.

2. Glanville DG, Mullineaux-Sanders C, Corcoran CJ, Burger BT, Imam S, Donohue TJ, et al. A High-Throughput Method for Identifying Novel Genes That Influence Metabolic Pathways Reveals New Iron and Heme Regulation in Pseudomonas aeruginosa. mSystems. 2021;6(1).

3. Halfmann A, Hakenbeck R, Brückner R. A new integrative reporter plasmid for Streptococcus pneumoniae. FEMS microbiology letters. 2007;268(2):217-24.

4. Glanville DG, Han L, Maule AF, Woodacre A, Thanki D, Abdullah IT, et al. RitR is an archetype for a novel family of redox sensors in the streptococci that has evolved from two-component response regulators and is required for pneumococcal colonization. PLoS pathogens. 2018;14(5):e1007052.
